# A Synthetic Community Approach Reveals Plant Genotypes Affecting the Phyllosphere Microbiota

**DOI:** 10.1371/journal.pgen.1004283

**Published:** 2014-04-17

**Authors:** Natacha Bodenhausen, Miriam Bortfeld-Miller, Martin Ackermann, Julia A. Vorholt

**Affiliations:** 1 Institute of Microbiology, ETH Zurich, Zurich, Switzerland; 2 Department of Environmental Sciences, ETH Zurich, Zurich, Switzerland; 3 Department of Environmental Microbiology, Eawag, Dubendorf, Switzerland; University of Wuerzburg, Germany

## Abstract

The identity of plant host genetic factors controlling the composition of the plant microbiota and the extent to which plant genes affect associated microbial populations is currently unknown. Here, we use a candidate gene approach to investigate host effects on the phyllosphere community composition and abundance. To reduce the environmental factors that might mask genetic factors, the model plant *Arabidopsis thaliana* was used in a gnotobiotic system and inoculated with a reduced complexity synthetic bacterial community composed of seven strains representing the most abundant phyla in the phyllosphere. From a panel of 55 plant mutants with alterations in the surface structure, cell wall, defense signaling, secondary metabolism, and pathogen recognition, a small number of single host mutations displayed an altered microbiota composition and/or abundance. Host alleles that resulted in the strongest perturbation of the microbiota relative to the wild-type were *lacs2* and *pec1*. These mutants affect cuticle formation and led to changes in community composition and an increased bacterial abundance relative to the wild-type plants, suggesting that different bacteria can benefit from a modified cuticle to different extents. Moreover, we identified *ein2*, which is involved in ethylene signaling, as a host factor modulating the community's composition. Finally, we found that different *Arabidopsis* accessions exhibited different communities, indicating that plant host genetic factors shape the associated microbiota, thus harboring significant potential for the identification of novel plant factors affecting the microbiota of the communities.

## Author Summary

The leaves of plants are inhabited by a diverse community of microorganisms. These leaf inhabitants influence their hosts with respect to growth and resistance to abiotic and biotic stresses. Recent studies revealed that the bacterial communities associated with leaves undergo selection, resulting in conserved microbial communities. However, the factors that are involved in structuring of bacterial communities are not well understood. In order to uncover host genetic factors that determine the community composition and to exclude confounding environmental effects, we inoculated *Arabidopsis thaliana* with a synthetic bacterial community under controlled conditions We screened a panel of Arabidopsis mutants defective in various traits for alterations in community structure and abundance and were able to show that cuticle synthesis and ethylene perception affect the bacterial community. In addition, we identified plant ecotypes with drastic differences in the community composition. Our system can thus be used to identify additional host genes and to broaden insights into plant microbe interactions, potentially providing a basis for applied plant protection through the identification of traits that enhance growth of plant probiotic bacteria.

## Introduction

The aerial parts of the plants, which are dominated by leaves, represent one of the largest terrestrial habitats for microorganisms [1]–[3]. This habitat, called the phyllosphere, is occupied by a diverse community of bacteria and fungi, which is important for plant health and growth [1]–[3]. Microorganisms in the phyllosphere can promote plant growth through the production of hormones. They can also be involved in plant protection, which is due to direct interactions of microorganisms through the production of antibiotic compounds and competition for resources [3]. Additionally, microorganisms may protect plants against pathogens by inducing systemic resistance [4], [5]. Commensals belonging to the genus *Sphingomonas* and their closely related species might represent part of the core phyllosphere community that protect plants against pathogens [6]. In addition, certain *Pseudomonas* strains have been shown to be plant protective agents [7].

Given the functional importance of the phyllosphere community on plant traits, it is relevant to understand the processes that are responsible for determining the composition of this community. This pertains to the fundamental question in community ecology of what principles underlie the assembly of strains into communities. A large body of theoretical and empirical work addresses this question (for a recent review see [8]), and has implied a vast number of different processes that play a role in community assembly [9] A recent synthesis groups this diversity of processes in just four classes – selection, drift, speciation and dispersal [10]. While this synthesis has not been specifically developed for microbial communities, it is well suited as a conceptual framework to describe and analyze the assembly of microbial communities [11]; furthermore, the suitability of the phyllosphere to test ecological concepts has been pointed out [12].

Here, we focus on selective factors that shape the assembly of the phyllosphere community, that is, on factors that have a consistent and reproducible effect on the composition of the microbial community on plant leaves. Previous studies have established that the bacterial phyllosphere communities are dominated by few phyla: Proteobacteria, Actinobacteria and Bacteroidetes [1], [13]. It is assumed that different factors contribute to the shaping of bacterial communities in the phyllosphere, including environmental cues, microbial interactions, the plant genotypes and phenotypes [1], and environmental factors such as temperature, water availability [14], [15], and geographic location (for example, [16]). The effects of plant factors on community composition have been demonstrated for leaf age [17], plant species [18], and cultivars [19]–[21]. Moreover, total population size is also affected by the plant species [22]. Several quantitative trait loci (QTL) have been identified as associated with bacterial diversity in corn [23] or with disease suppression in tomato [24]. However, no direct effects of specific genes on the composition of the phyllosphere community could be established in these studies. Using the model plant *Arabidopsis thaliana*, one study identified jasmonic acid synthesis as a factor driving epiphytic diversity in the phyllosphere [25], whereas another study did not reveal any effect for trichomes on bacterial diversity [26].

There are a number of plant factors that could potentially have selective effects on phyllosphere microbial communities. A first potentially important factor is the hosts' innate immune system. Plants recognize bacteria at two levels of their immune system: the first level is pattern-triggered immunity (PTI), whereby plant receptors recognize microbial-associated molecular patterns (MAMPS), for example, flagellin [27]; and the second level is effector-triggered immunity (ETI), where intracellular plant receptors recognize microbial effectors, which are virulence factors transferred by pathogens into the host cytoplasm to dampen PTI [28]. It is not known how plants discriminate between pathogenic and commensal or beneficial microorganisms and whether plant receptors recognize these non-pathogenic phyllosphere bacteria and trigger plant immune signaling networks downstream of PTI or ETI activation, with potential effects on community structure. The habitat is scarce in nutrients [1] so other potential traits that may influence the presence of plant-associated microorganisms include, for example, mutants in sugar transporters [29] or amino acid transporter [30]. Similarly, it is not known whether mutants defective in secondary metabolites used for defense, such as camalexin, glucosinolates [31], and flavonoids [32], affect their associated microbial populations. More specifically, mutants in pectin synthesis are hypothesized to affect the abundance of methylotrophic bacteria because methanol, as a by-product of pectin synthesis, is an important factor for bacterial growth under competitive conditions [1], [33].

In general, identifying host genetic factors using field experiments is challenging because of the confounding influence of the external environment as well as the diversity of natural microbial communities. To reduce environmental complexity, gnotobiotic model systems with well-defined communities represent an alternative approach. The advantages of such controlled systems are that they allow for reproducible experimentation and the use of molecular fingerprinting methods to characterize the defined community. In mice, bacterial synthetic communities have been successfully used to study how diet impacts the microbiota [34], [35]. To our knowledge, synthetic bacterial communities have not yet been used to identify plant host genotypes that shape the associated microbiota.

Using a synthetic community approach, we aimed to identify plant genetic factors that influence community composition and/or the bacterial abundance of the leaf-associated community of *A. thaliana*. A set of 55 plant mutants was screened for such phenotypic effects, resulting in the identification of three mutants with significant community alterations. In addition, of the nine natural accessions tested, four were found to modify community composition and abundance, indicating that natural variation can be used for future experiments with the synthetic community to identify novel host genes affecting phyllosphere microbiota.

## Results

### Establishment of a core synthetic community of the *A. thaliana* Col-0 phyllosphere

Knowledge of the overall composition of the microbiota of the *A. thaliana* phyllosphere [1], [13], [36] provides invaluable information for formulating a core microbiota based on cultivated model strains. A laboratory strain collection was used to establish a bacterial synthetic community, which allowed for the reproducible colonization of the phyllosphere in a gnotobiotic system. First, 20 strains were tested as individual inoculates. To be included in the synthetic community, strains were chosen that met two criteria: i) they were able to colonize the phyllosphere (higher than 10^7^ CFU/g leaf fresh weight upon single inoculation, see Table 1), and ii) they did not induce disease symptoms nor cause a reduction in growth. In addition, the strains needed to represent the most abundant phylogenetic groups detected in the phyllosphere. Because Alphaproteobacteria is the most abundant sub-phylum in the phyllosphere of *A. thaliana* [1], four species were selected to represent this phylogenetic group: *Sphingomonas phyllosphaerae* and *Sphingomonas* sp. Fr1, which both have a plant-protective effect on *A. thaliana* [6], and *Methylobacterium radiotolerans* and *M. extorquens* PA1, which are efficient colonizers of the phyllosphere [37] but do not show a plant-protective effect [6]. In addition, two representatives of the Actinobacteria and one Betaproteobacteria were chosen for these abundant phyla of the phyllosphere (Table 1). Although five different strains of Gammaproteobacteria were tested as single isolates (Table S1), none could be included in the community because those strains either reduced plant growth or induced a strong disease phenotype under the experimental conditions. Mixing these strains with the rest of the community did not mask the disease phenotype.

**Table 1:** Bacterial strains to build the synthetic community used in this study.

Strain	Phylum	Reference	Representative ARISA Peak (±2–3 pb)	Mean population size log cfu/gFW (± SE)	Relative fluorescence intensity (%)^a^	Numbers of 16S rRNA gene copies
*Methylobacterium extorquens* PA1	Alphaproteobacteria	[37]	845	7.8 (0.092) d	4.6±2.0	5^c^
*Methylobacterium radiotolerans* 0-1T (DSM 1819)	Alphaproteobacteria	[78]	625	[37]	11.6±2.9	5^c^
*Sphingomonas* sp. Fr1	Alphaproteobacteria	[6]	905	8.1 (0.15)^d^	22.9±3.9	3^b^
*Sphingomonas phyllosphaerae* (DSM 17258T)	Alphaproteobacteria	[79]	910	[6]	3±0.8	3^b^
*Variovorax* sp. #613	Betaproteobacteria	Laboratory collection*	760	8.7 (0.14)^d^	9.0±2.7	1^b^
*Arthrobacter* sp. #968	Actinobacteria	Laboratory collection*	735	7.5 (0.12)^d^	8.7±1.5	5^b^
*Rhodococcus* sp. #964	Actinobacteria	Laboratory collection*	505	7.3 (0.11)^d^	40.1±3.2	5^b^

* isolated by C. Knief from *M. truncatula* (#613) and *A. thaliana* (#964 and #968).

a Relative fluorescence intensity determined by ARISA of the community colonizing Col-0 plants two weeks after inoculation (weighted mean ± weighted standard deviation, 10 biological replicates).

b 16S rRNA gene copy number determined by Southern blot analysis.

c 16S rRNA gene copy number based on the genome sequence.

d One-week old plants were inoculated with single isolates and mean population was estimated two weeks post-inoculation (n = 6 plants).

To monitor changes in community composition in a high-throughput manner, automated ribosomal intergenic spacer analysis (ARISA) was used. Briefly, the 16S–23S rRNA intergenic spacer region was amplified by PCR using fluorescence-tagged universal primers. The PCR products were separated using a capillary sequencer. Each species in the community could be distinguished from the others based on its unique ARISA profile. Because some species were characterized by multiple peaks due to multiple 16S rRNA gene copies and variable length of the intergenic spacer regions, one representative peak was chosen for each species (Table 1). In addition, the peak area was normalized by the 16S rRNA gene copy number so that the abundance of each species was roughly proportional to the peak area in a semi-quantitative approach [38], [39] (for a validation experiment in which the DNA of one species was diluted against a mixture of DNA background see Figure S1).

A time course experiment was performed where the seven-member synthetic community (Table 1) was assessed immediately after spray inoculation of wild-type Col-0 plants and once a week for four weeks thereafter. Community composition was compared using the Bray-Curtis dissimilarity index (the more different two communities are, the closer to 1 their index is). Figure S2A shows that community comparisons of the inoculum to leaves sampled immediately after spraying were indistinguishable from community comparisons of leaf samples with each other (*P*>0.05). After one week, community comparisons of plant samples to inoculation solution was significantly greater relative to community comparisons made within plant samples (*P* = 0.0048). Paralleled determination of the population sizes by leaf washings revealed that the number of bacteria per plant was 1.18*10^3^ immediately after spraying and increased steadily through time (Figure S2B). Based on the time-course experiment, we decided for the remainder of the study to harvest the leaves two weeks post-inoculation to allow growth of and competition between bacteria.

Using ARISA to analyze the community associated with wild-type Col-0 samples from ten independent biological experiments, we found that the synthetic community colonizes the phyllosphere in a reproducible manner (Figure S3). The average relative fluorescence intensity after colonization ranged from 3% for *S. phyllosphaerae* to 40% for *Rhodococcus* (Table 1).

A real-time qPCR method was developed to estimate the bacterial abundance in the phyllosphere. First, we confirmed that the PCR primers amplify the 16S rRNA gene in a linear fashion in the absence (Figure S4A) and in the presence of plant DNA (Figure S4B). The relative abundance of the 16S rRNA gene was calculated by normalizing with a plant gene and is proportional to the amount of bacterial DNA (Figure S4C).

### Screening *Arabidopsis* genotypes

**Figure 1:**
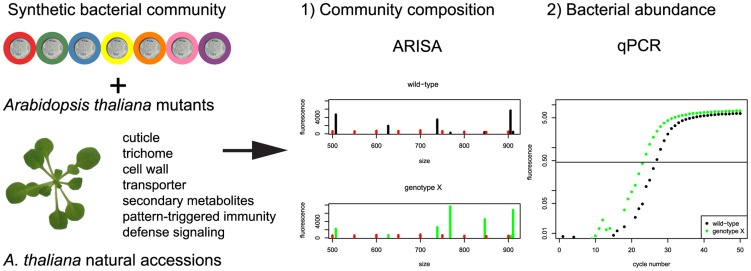
Experimental strategy to identify the plant genes responsible for changes in community composition and/or total bacterial abundance. See text for details.

To identify plant host genetic factors that influence community composition, *a priori* candidate genes were selected from six different classes: cuticle and trichome, cell wall and pectin synthesis, secondary metabolism, sugar and amino acid transporters, defense signaling and pattern-triggered immunity (see Table S2 for a complete list of mutants). In addition, a small panel of natural accessions was screened. For each plant genotype, community composition and the 16S rRNA gene copy number were assessed (Figure 1).

**Figure 2:**
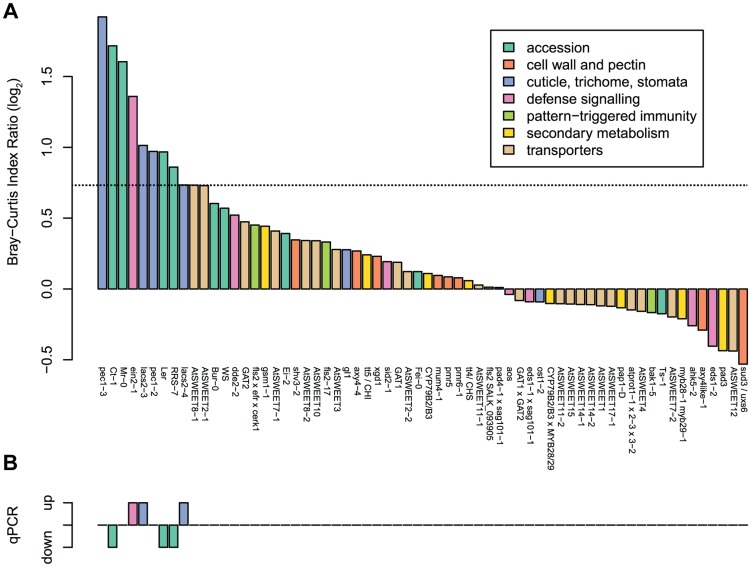
Different *A. thaliana* plant genotypes (55 in total) were tested for changes in bacterial community composition and abundance in independent experiments. (a) Results of the ARISA analysis. The average Bray-Curtis index was calculated for each genotype compared to the wild-type in pairwise comparisons and divided by the average Bray-Curtis index for each genotype with itself (see Figure S7 for individual values). Col-0 was used to normalize for most genotypes, except for mutants with a different background, which were normalized to their respective background. The horizontal dashed line indicates the limit of reproducibility of the system, mutants very close to this threshold could either be confirmed (Figure 3) or not (Figure S8). (b) Results of the qPCR analysis. The up and down bars indicate higher and lower 16S rRNA gene copy numbers, respectively, compared to the wild-type Col-0 samples (Bonferroni-adjusted *P* value <0.05). For each genotype there were 3–4 DNA pools from five plants. The same DNA extracts were used for ARISA and qPCR analysis.

A total of 55 *A. thaliana* mutants and accessions were tested in 10 independent experiments, each including Col-0 as a control and Landsberg erecta (Ler) as well as Wassilewskija (WS) when needed, dependent on the genetic background of each mutant. Results are shown in Figure S5 for individual screens and Figure S6 for all plant genotypes as a clustering analysis based on the Bray-Curtis dissimilarity index. We observed that the communities from some of the selected mutants clustered together and separate from Col-0. To compare independent experiments, community comparisons of each genotype to wild-type samples were calculated with the Bray-Curtis Dissimilarity index (Figure S7). Col-0 was used as the wild-type, except for mutants with a different background. In order to exclude genotypes where samples showed high variability from each other, community comparisons were also made within plant samples. Figure 2A shows the ratio of comparisons between each genotype and the corresponding wild-type over the comparison within each genotype. For each of these experiments, the 16S rRNA gene copy number was determined by qPCR (Figure 2B).

From the initial screen, the ten plant genotypes showing the highest dissimilarity to the wild-type (Figure 2A) and the six ecotypes with higher or lower bacterial abundances (Figure 2B) were selected for validation experiments using ARISA and qPCR. In addition, colony forming units were determined to verify altered community abundances and compositions using a cultivation-dependent method. From these sets of genotypes, 3 mutants (*lacs2*, *pec1*, and *ein2*) and 4 accessions (Mr-0, Ler, RRS-7, and Ct-1) with an altered community composition and/or overall abundance could be verified (see below). In contrast, results with the ‘sweet’ mutants could not be confirmed in validation experiments, and no genotype effect for community composition nor for bacterial abundance was observed (Figure S8).

### Cuticle synthesis is an important factor for community composition and abundance

**Figure 3:**
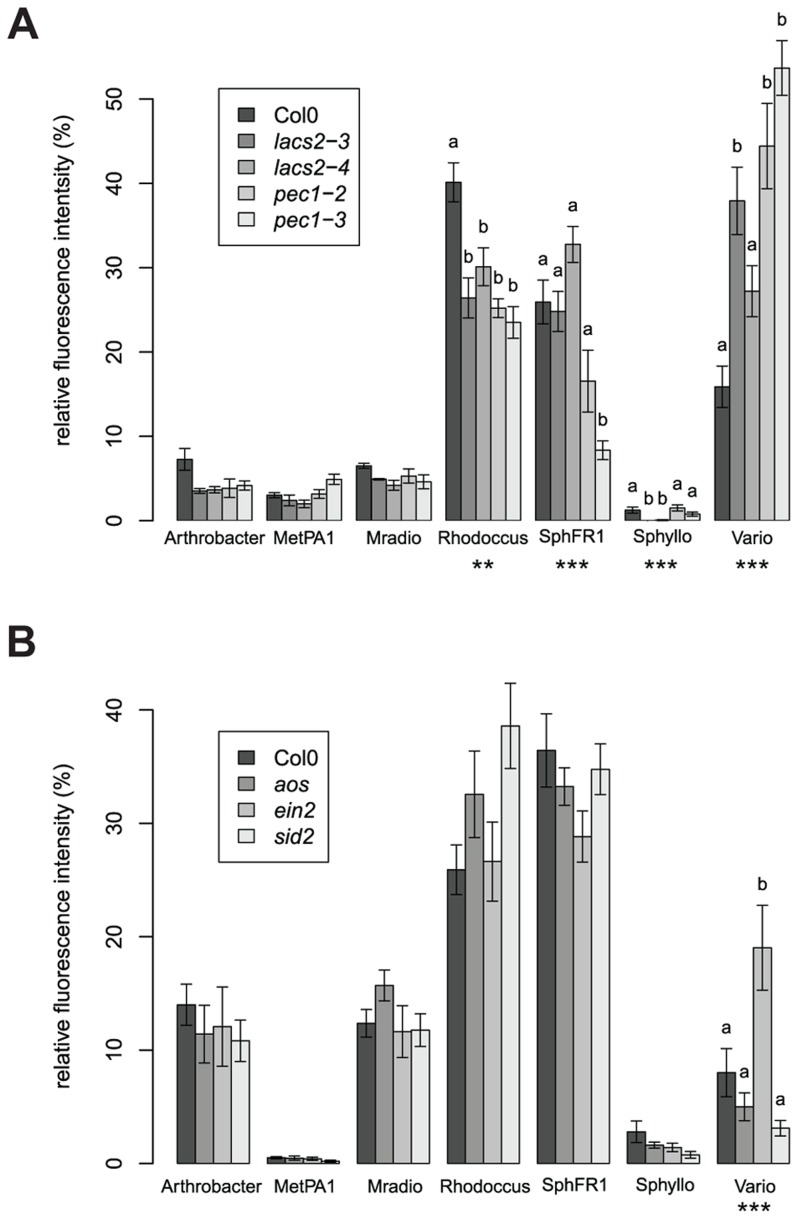
Different community compositions were detected on the cuticle and ethylene mutants. (a) Cuticle mutants (b) Defense signaling mutants. Average (± s.e.m) relative fluorescence intensity (RFI). Asterisks indicate a significant plant genotype effect for the RFI of the bacterial species (*, *P*<0.05; **, *P*<0.01, ***, *P*<0.001; Bonferroni-adjusted *P* values). Letters indicate a significant effect of the genotype compared to Col-0 (*P*<0.05, Dunnet's test). There were four (a) and six (b) DNA pools from five plants for each genotype. These experiments were repeated at least in triplicate with similar results. Bacterial species abbreviations: Arthrobacter = *Arthrobacter* sp. #968, MetPA1 = *Methylobacterium extorquens* PA1, Mradio = *Methylobacterium radiotolerans* 0-1T, Rhodoccus = *Rhodococcus* sp., SphFR1 = *Sphingomonas* sp. Fr1, Sphyllo = *Sphingomonas phyllosphaerae*, Vario = *Variovorax* sp.

**Figure 4:**
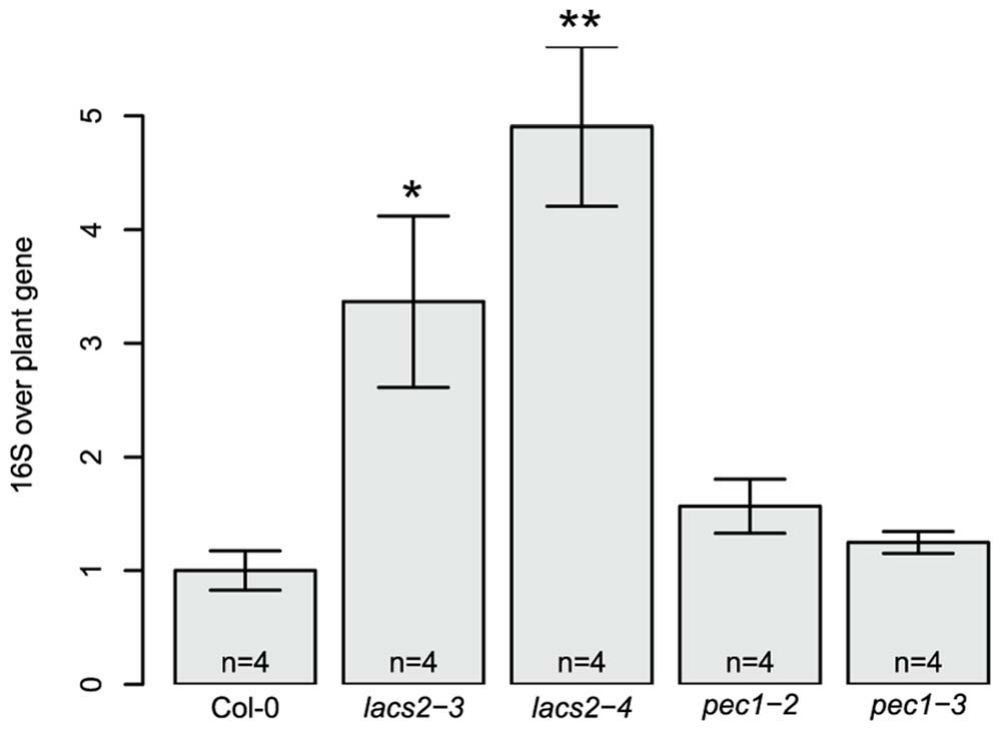
Higher 16S rRNA gene copy numbers were found for the lacs2 mutant compared to the wild-type plants. The number of 16S rRNA gene copies was normalized using a plant gene AT4G33380 and normalized to the wild-type. The number of DNA pools analyzed for each genotype is indicated in the barplot. Asterisks indicate a significant effect for genotype compared to Col-0 (*, *P*<0.05; **, *P*<0.01; Student's t-test, Bonferroni- adjusted *P* values). These experiments were repeated at least in triplicate with similar results.

A different community composition of *lacs2* and *pec1* samples was confirmed with independent replicate experiments using two independent alleles each (Figure 3A). Multivariate analysis of variance confirmed a significant effect of these genotypes across replicate experiments (Table 2 and Table S3). *LACS2* encodes for long-chain acyl-coenzyme A synthetase 2, an enzyme involved in cutin biosynthesis. The mutant plants are characterized by the absence of the cuticular membrane and a reduction of cuticular polyesters, which normally compose the wild-type cuticle [40]. The *pec1* mutant has an intermediate phenotype between Col-0 and *lacs2* and carries a mutation in an ATP-binding cassette transporter involved in the export of cuticle precursors [41]. Statistical tests indicated that there was a genotype effect for the relative fluorescence intensity (RFI) of *Rhodococcus*, *Sphingomonas* sp. Fr1, *S. phyllosphaerae* and *Variovorax*. Compared to the wild-type, both mutants harbored more *Variovorax* and less *Rhodococcus*, whereas *pec1-3* had less *Sphingomonas* sp. Fr1. Using the qPCR method to estimate relative 16S rRNA gene copy numbers, we found that *lacs2* harbored a higher bacterial abundance compared to the wild-type (Figure 2B). Multivariate analysis of variance indicated that there is a genotype effect for 16S rRNA gene copy numbers of both lacs2 and pec1 (Table 2 and Table S4) which was further confirmed by the fact that both *lacs2* alleles carried a higher bacterial abundance compared to wild-type plants (Figure 4).

**Table 2:** Multivariate analysis of variance for community composition and bacterial abundance.

ADONIS^a^	lacs2-3	pec1-3	ein2	Ct-1	Ler	Mr-0	RRS-7
Experiment	0.021	0.002	0.001	0.001	0.001	0.001	0.001
Experiment*genotype	0.110	0.060	0.601	0.106	0.001	0.001	0.002
Genotype across experiment	0.001*	0.001*	0.001*	0.001*	0.001*	0.001*	0.001*
**ANOVA^b^**	**lacs2-3**	**pec1-3**	**ein2**	**Ct-1**	**Ler**	**Mr-0**	**RRS-7**
Experiment	0.8926	0.03544	0.65150	0.7120	0.08908	5.731e-05	0.1398
Experiment*genotype	0.8640	0.06663	0.46089	0.6932	0.13805	5.889e-05	0.1505
Genotype across experiment	2.935e-06*	5.085e-05*	0.00518*	2.711e-06*	1.033e-07*	4.802e-10*	2.099e-09*

a Values shown are the P values resulting from analysis of variance using distance matrices (adonis) of community composition associated with leaves of Col0 and the indicated genotype in the replicate experiments (3 or 4 experiments depending on the genotype).

b Values shown are the P values resulting from analysis of variance (ANOVA) of the 16S rRNA gene copy numbers of Col0 and the indicated genotype in the replicate experiments (3 or 4 experiments depending on the genotype). Data were first normalized to the wild-type and then log-transformed.

Asterisks mark tests for ‘Genotype’ that are significant after Bonferroni correction for multiple testing (seven independent tests).

The results of the ARISA and qPCR analysis were partially confirmed using bacterial enumeration (Figure S9). For example, *Variovorax* cells were more abundant on the *lacs2* and *pec1* mutants, in-line the ARISA results. Contrary to the ARISA results, *Sphingomonas* were more abundant on both the *pec1* and *lacs2* mutants, suggesting that the relative abundance (ARISA results) does not necessarily reflect the absolute abundance (bacterial enumeration). This discrepancy might be due to differences in the protocols. ARISA profiles were determined using DNA extracted from the whole plants (both epiphytic and endophytic communities), whereas the protocol for leaf enumeration possibly extracts more epiphytic than endophytic bacteria.

### Ethylene signaling is a factor involved in community composition

Independent replicate experiments confirmed a shift in the synthetic bacterial community colonizing the *ein2* mutant plants compared to the wild-type plants (Figure 3B). The adonis test validated a significant effect of genotype across replicate experiments (Table 2 and Table S3). There was a plant genotype effect for the RFI of *Variovorax*, which was higher on the *ein2* mutant. The RFI of other bacterial species were not affected by plant genotype. The *ein2* mutant is ethylene insensitive [42] and carries a mutation in EIN2 [43], which plays a central role in the ethylene response, an important hormone for response to the environment and plant defense [44]. On the contrary, when we tested other mutants in the plant defense signaling pathways, we found that the jasmonate mutant *aos* and the salicylic acid mutant *sid2* harbored a similar community composition compared to the wild-type plants (Figure 3B). In the initial screen, bacterial abundance, as measured by relative 16S rRNA gene copy number, was found to be higher on the *ein2* mutant (Figure 2B). ANOVA confirmed a significant effect for this genotype (Table 2 and Table S4); however, this effect was weak and not significant for a single experiment (Figure S10). The ARISA results were confirmed using bacterial enumeration (Figure S9). *Variovorax* cells were more abundant on the *ein2* mutant, whereas the abundance of other bacterial species was not affected by this mutation.

### Different *Arabidopsis* accessions harbor different communities

**Figure 5:**
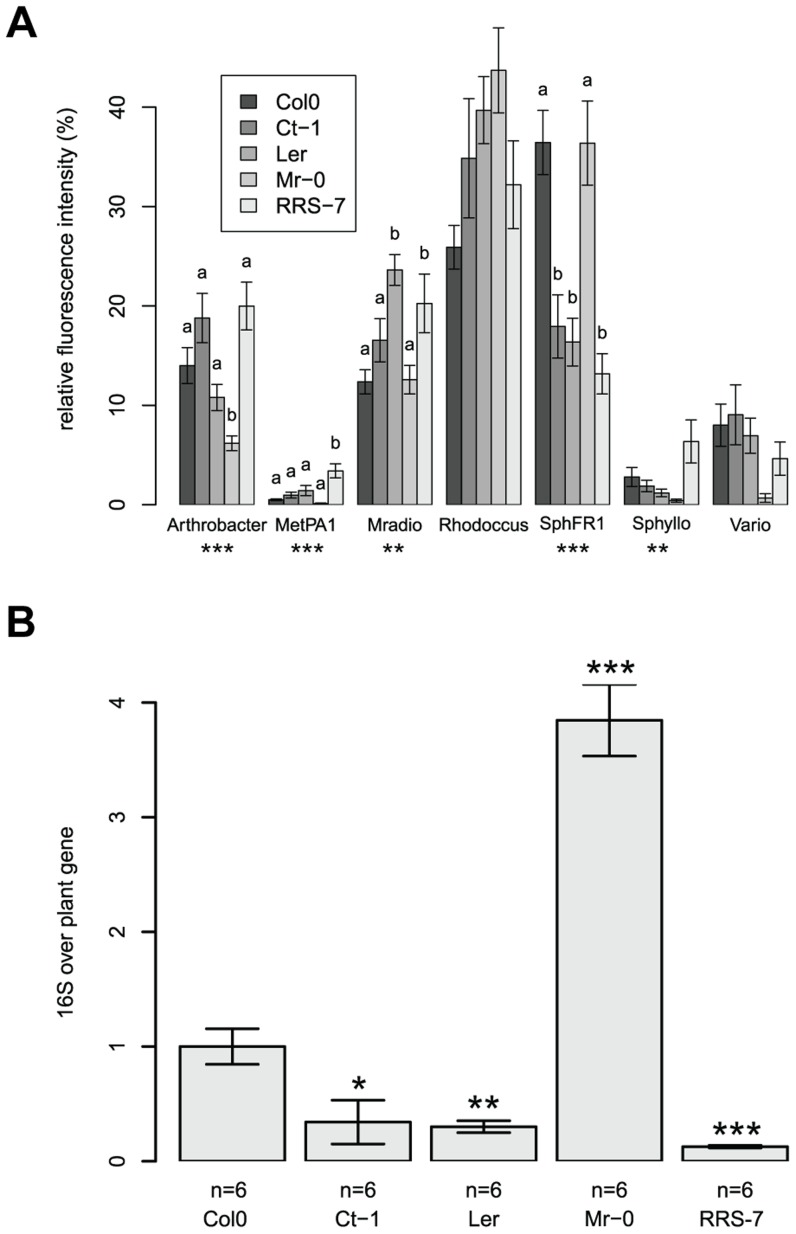
Natural variation in the host has an effect on its associated bacterial communities. (a) Community composition determined by ARISA. Average relative fluorescence intensity (± s.e.m). For abbreviations see Figure 3. Letters indicate a significant effect of the genotype compared to Col-0 (*P*<0.05, Dunnet's test). (b) 16S rRNA gene copy number. Asterisks indicate a significant effect of genotype compared to Col-0 (*, *P*<0.05; **, *P*<0.01; ***, *P*<0.001; Bonferroni-adjusted *P* values). There were 6 DNA pools from five plants for each genotype. These experiments were repeated in triplicate with similar results.

The four *Arabidopsis* accessions identified in the first round of screening with a different community composition, Mr-0, Ler, Ct-1, and RRS-7, were confirmed in independent replicate experiments (Figure 5A). Multivariate analysis of variance confirmed a significant effect of each genotype on community composition (Table 2 and Table S3). Statistical tests revealed that there was a genotype effect for the RFI of *Arthrobacter* (lower in Mr-0), *M. extorquens* PA1 (higher in RRS-7), *M. radiotolerans* (higher in Ler and RRs-7), and *Sphingomonas* sp. Fr1 (lower in Ct-1, Ler and RRS-7. In addition, bacterial abundance was also different on the natural accessions as indicated by quantitative qPCR of the 16S rRNA gene (Table 2 and Table S4). The 16S rRNA gene copy numbers were higher on Mr-0 and lower on Ler, Ct-1, and RRS-7 (Figure 5B).

## Discussion

In this study, we established a synthetic community approach to examine the effect of host genotype on the bacterial community composition and total abundance on plant leaves. We demonstrate that a model microbiota developed in a reproducible manner in the phyllosphere, allowing for the monitoring of perturbations dependent on the host's genotype. This system has several advantages, including its relatively short time scale and small space requirements, facilitating the independent biological repetition of experiments. Although in the future, more complex communities might be tested to link plant genotype to bacterial abundance, the low complexity community applied in this study has already allowed for the identification of three *A. thaliana* mutants with effects on community composition and/or total abundance. The plant mutants with the strongest effect on the associated bacteria were *lacs2* and *pec1*, which are characterized by a more permeable cuticle compared to wild-type plants [40], [41], [45]. The cuticle is present on the outside surface of epidermal cells and is composed of the aliphatic polyester cutin and waxes [46]. In addition to its function as a diffusion barrier that diminishes water loss and as a protection against abiotic stresses, such as UV radiation [47], the cuticle also serves as a key interface for plant-microbe interactions. First, the cuticle represents the initial interaction surface with microorganisms colonizing the phyllosphere, therefore, features of the cuticle affect adhesion and, thus, microbial immigration [48]. Second, the cuticle controls transpiration, thus reducing water availability, which is a limiting factor for the growth of phyllosphere bacteria [15]. Third, the cuticle is involved in the transport of polar solutes and lipophilic organic compounds, thus reducing nutrient availability [47]. Furthermore, the question of whether components of the cuticle themselves can be used as substrate by bacteria is open. Evidence for the importance of the cuticle for the phyllosphere community comes from both sides of the study of plant-bacteria interactions. In terms of bacteria, epiphytic bacteria have been shown to alter the leaf surface permeability of isolated intact cuticles [49]. In terms of the plants, epicuticular wax synthesis has been shown to impact the colonization of single bacteria inoculates using maize mutant plants [50], and to impact the phyllosphere community composition, as shown very recently in Arabidopsis [51]. Moreover, epidermal thickness was correlated with the bacterial population size colonizing eight Mediterranean plant species [52].

In this study, we present evidence that cuticle permeability affects not only bacterial abundance but also community composition. We observed that *lacs2* and *pec1* mutants harbored more *Variovorax*. The average RFI of *Variovorax* in Col-0 was 16%; however, for the cuticle mutants, the average RFI ranged from 27% (*lacs2-4*) up to 54% (*pec1-3*). One explanation is that an enhanced permeability of the cuticle leads to an increase in nutrient availability, which favors the growth of the Betaproteobacterium *Variovorax*, the name of which notably refers to its ability to consume many different substrates. In contrast, the RFI of *Rhodococcus* decreased for both cuticle mutants, ranging from 23% (*pec1-3*) to 30% (*lacs2-4*) compared to 40% in Col-0. One possible explanation is that *Rhodococcus* feeds on cuticle components, which are less abundant in the mutants. In contrast, the abundance of both *Methylobacterium* strains was not different on the cuticle mutants, which might indicate that methanol availability is not affected by either mutation.

We found that the *lacs2* mutant carried a higher bacterial abundance compared to both the wild-type and *pec1* mutant (Figure 4). The *pec1* mutant was shown to have an intermediate phenotype to *lacs2* in terms of cuticular permeability, as measured using toluidine blue staining, sensitivity to herbicides and water loss [41]. Moreover, analysis of the cuticle ultrastructure revealed that *pec1* retains a thick layer of electron-dense material, representing insoluble lipid-derived polymers, that is missing in *lacs2* [40]. Analyses of the leaf polyester monomers demonstrated that the monomer composition of the *lacs2* mutant was reduced by 20–25% compared to wild-type amounts [40], whereas only minor changes were observed for *pec1* [41]. Interestingly, both *lacs2* and *pec1* mutants are more resistant to the fungal pathogen *Botrytis cinerea*, with the increased resistance proposed to be due to the induction of antifungal compounds by elicitors diffusing through the cuticle [40]. In contrast, *lacs2* is more susceptible to avirulent *Pseudomonas syringae*, with the increased susceptibility hypothesized to be due to enhanced tissue collapse upon infiltration of the pathogen [53]. However, tissue collapse likely does not play a role in our study because the synthetic community was not syringe-infiltrated but rather sprayed onto the leaves. Therefore, we hypothesize that the higher bacterial abundance phenotype measured on *lacs2* was due to the increased leaching of nutrients from this mutant compared to the wild-type and *pec1* mutant.

The ethylene-insensitive mutant *ein2* harbored a different community composition. In particular, *Variovorax* was more abundant in the phyllosphere of the mutant compared to the wild-type plants. Ethylene is a plant hormone with multiple roles in development, such as seed germination, fruit ripening, and root hair formation [54], [55]. In addition, ethylene modulates plant resistance to pathogens, which is dependent on the type of attacker. Generally, ethylene is found to reduce the appearance of diseases caused by necrotrophic and hemibiotrophic pathogens and to increase disease symptoms caused by other types of pathogens [56]. Interestingly, several pathogenic bacteria and fungi interfere with the plant defense-signaling pathway by producing ethylene [57], [58], which, in this case, can be considered a virulence factor. In contrast, some plant growth-promoting bacteria colonizing roots can degrade the compound 1-aminocyclopropane-1-carboxylic acid (ACC), the precursor of ethylene, using the enzyme ACC deaminase, thereby increasing root length [59]. In this study, we found the phyllosphere of the *ein2* mutant to show quantitative differences in the community composition. In the *A. thaliana* rhizosphere, the *ein2* mutant was found to harbor a lower bacterial abundance and was not associated with any changes in bacterial community composition [60]. In contrast, in the tobacco rhizosphere, Long et al. found a lower bacterial diversity and a different community in ethylene-insensitive transgenic plants compared to wild-type plants [61]. The distinct functions of ethylene in roots and leaves might affect the associated communities differently, for example, ethylene is involved in the formation of root hairs, which are hypothesized to serve as an entry point for the bacterial colonization of roots. Wild-type tobacco plants have also been demonstrated to have more root hairs compared to ethylene-insensitive plants and have been found to be associated with a different bacterial community [61].

Natural accessions of *A. thaliana* are a source of genetic diversity that can be harnessed to identify novel genes underlying phenotypic variations, which can then be used for quantitative trail locus (QTL) analyses of recombinant inbred lines (RIL) [62]. Furthermore, the recent development of cheaper SNP arrays and sequencing technology has enabled genome wide associations (GWA) in *A. thaliana* [63]. Natural accessions thus provide a valuable resource to begin identifying the intricate relationship of plants and associated microorganisms. Recently, using 16S rRNA gene amplicon sequencing of root samples and analyzing eight *Arabidopsis* accessions, Lundberg et al. [64] identified a small subset of 12 operational taxonomic units (OTU) out of 778 that showed host genotype dependent quantitative differences. In another study only one OTU of the root endophyte community showed significantly different quantitative enrichment when analyzing two *Arabidopsis* accessions [65]. Field and sample types (rhizosphere versus bulk soil) were found to be more important than the plant genotype for bacterial root community composition [65], [66].

Notably, here using a synthetic community approach applied to the phyllosphere of Arabidopsis we found that 4 out of the 9 accessions tested harbor a different community composition compared to Col-0, indicating that natural accessions offer significant potential for the discovery of new genes affecting community composition and/or abundance. In addition, several of the tested accessions harbor different *Methylobacterium* and *Arthrobacter* abundances that were not affected by the *lacs2*, *pec1* and *ein2* mutants. Interestingly, we found accessions with both lower (Ct-1, Ler, and RRS-7) and higher (Mr-0) bacterial abundances compared to the Col-0 accession. Future experiments with the model synthetic community and methods developed in this study will represent a valuable approach to map and identify novel genes affecting community composition and bacterial abundance.

## Materials and Methods

### Bacterial strains

The isolates and type strains used for the synthetic community are listed in Table 1. *Variovorax* sp., *Arthrobacter* sp. and *Rhodococcus* sp. were isolated from wild plants growing at different sites located near Madrid, Spain and described in [16]. The 16S rRNA genes of these three strains were sequenced for verification. The 16S rRNA gene copy number was determined by Southern blot.

### Plant growth conditions

*A. thaliana* plants were cultivated on half-strength MS nutrient medium including vitamins and 0.55% plant agar (both from Duchefa, Haarlem, Netherlands) and supplemented with 1% sucrose. Seventy milliliters of medium was poured into microboxes outfitted with a XXL filter (Combiness, Nazareth, Belgium). To avoid leaves touching the medium, a sterile Lumox Film 25 (Sarstedt, Nümbrecht, Germany) with 6 holes (diameter, 4 mm) was placed on the agar surface. *A. thaliana* seeds were surface sterilized using a standard protocol [6] and stratified for 3 days (at 4°C) before being placed at the holes. Plants were grown under short-day conditions (a 9-h photoperiod) in a standard growth chamber, as previously described [6].

### Plant inoculation

Both *Methylobacterium* strains were grown on mineral salt medium [67] supplemented with 0.5% succinate as a carbon source. All other strains were grown on nutrient broth (NB) without additional NaCl (Sigma-Aldrich, St. Louis, MO, USA). The two *Sphingomonas* strains were grown in liquid cultures, whereas the other strains were grown on solid media. Strains were grown at 28°C for three days (both *Methylobacterium* strains), two days (*Rhodococcus* sp., *Variovorax* sp., *Arthrobacter* sp. and *S. phyllosphaerae*), or one day (*Sphingomonas* sp. Fr1). Before inoculation, cells from the liquid cultures were washed once and resuspended in 10 mM MgCl_2_ solution. For cultures grown on solid medium, a loop of material was resuspended in 10 mM MgCl_2_ solution. The optical density at 600 nm (OD600) of each solution was adjusted to 0.2. The synthetic community was obtained by mixing the seven strains at 1∶1∶1∶1∶1∶1∶1 OD600. This solution was then diluted to an OD600 of 0.02. Plants were inoculated by spraying 200 µl of bacterial suspension with an airbrush paint gun [68].

### Harvesting

For the time course experiment, samples were harvested immediately after spraying (four DNA pools, each with ten plants), and 1, 2, 3, and 4 weeks after inoculation (for each time point, four DNA pools, each with five plants). For the screening of plant genotypes, plants were harvested two weeks after inoculation (five plants from five different microboxes were pooled for one DNA extraction). In total, between 3 and 6 DNA pools per genotype were sampled depending on the experiment. Plants were taken out of the microboxes, and the roots and cotyledons were removed using flame-sterilized scalpels and forceps. In addition, for the validation experiments, the population size colonizing individual plants was determined using a dilution series (see below for the protocol).

### DNA extraction

DNA was extracted from the plant tissues using the NucleoSpin Plant II kit (Macherey-Nagel, Düren, Germany). Plant samples were lyophilized, and one metal bead was added to each sample in a 2 ml- centrifuge tube before chilling in liquid nitrogen. Samples were homogenized for 2 min at 25 Hz using a Retsch TissueLyser (Retsch, Haan, Germany). The SDS-based lysis buffer PL2 was added to the homogenized samples, and the standard protocol according to the manifacturer's instructions was used thereafter.

### ARISA

Primer 1492F (reverse complement of 1492R, [69]), and 23Sr [70] were used to amplify the 16S–23S rRNA intergenic space region. These primers were tested with DNA from plants grown axenically; no product was detected, indicating that they do not amplify mitochondrial or chloroplast DNA. Primer 1492F was labeled at the 5′ end with fluorescein. The reaction volume was 25 µl and contained 1-fold Phusion HF reaction buffer, 200 µM dNTP, 250 nM of each primer, 3% DMSO, 0.4 units of Phusion polymerase and approximately 20 ng DNA. The PCR program consisted of an initial denaturation step of 4 min at 94°C, followed by 35 cycles of denaturation at 94°C for 30 sec, annealing at 60°C for 30 sec, elongation at 72°C for 1 min followed by a final elongation step at 72°C for 7 min. PCR products were verified on agarose gels before preparing for ARISA. PCR products (2 µl, diluted 10- and 20-fold) were mixed with 8 µl of HiDi formamide (Applied Biosystems) and 0.2 µl MapMarker 1000-ROX (BioVentures, Murfreesboro, USA). After denaturation at 95°C for five minutes, the samples were analyzed using a 3130 ABI capillary sequencer. Genemapper version 3.7 (Applied Biosystems) was used for the data analysis. Sizing tables were exported for analysis with R.

### qPCR

The reaction volumes were 20 µl and contained 1-fold FastStart Universal SYBR green Master (Roche Applied Science), UltraPure DNase/RNase-free water (Life Technologies), 600 nM primer mix (16S rRNA primers) or 300 nM (plant gene primers), and approximately 5 ng DNA. The 16S rRNA gene was amplified using primers 799F [71] and 904R [72], see Table S5 for the primer sequences. Although these primers were designed to exclude organelle DNA, a product is amplified with DNA from plants grown axenically (deltaCt = 6–9 between inoculated Col-0 plants and axenically grown plants). This PCR product was cloned and sequenced and found to include sequences from organelles (chloroplast and mitochondria) confirming that the plants were axenic. Because we compared the samples with each other, we assumed that the small amount of plant DNA amplified by those primers did not affect our estimations. The primers ExpF1 and ExpF2 (Table S5) amplify the plant gene AT4G33380, a reference gene used for transcript normalization [73], which was used to normalize the 16S rRNA gene copy number. Controls, including no template, were included for each run. PCR assays were run in duplicate on a Rotorgene 3000 (Corbett Life Science, Qiagen). The PCR program consisted of a touchdown program with an initial denaturation step of 10 min at 95°C, followed by 35 cycles of 15 sec of denaturation at 95°C, 25 sec for the annealing step with the temperature decreasing from 65°C to 55°C (2 degrees per cycle), and 45 sec of elongation at 72°C followed by a melting curve analysis.: The raw data were exported directly from Corbett Research Software version 1.7 and imported into LinRegPCR version 12.8 [74] to determine cycle number to threshold (Ct) and efficiency (E). The 16S rRNA gene copy number was normalized to the plant gene AT4G33380 and calculated as follows: 16S rRNA/plant gene = *E_plant gene_* ^Ct plant gene^/*E_16S_* ^Ct 16S^, where Ct is the mean of the 2 duplicate reactions and E is the mean for all reactions with a particular primer pair for each run. To test for linearity of the qPCR method, a two-fold dilution series of *Variovorax* DNA was prepared (starting concentration 1 ng/µl DNA). qPCR was run following the standard protocol with 5 µl of bacterial DNA in the absence (Figure S4A) or presence of plant DNA (5 ng per reaction) (Figure S4B).

### Enumeration of phyllosphere bacteria

Cell numbers were determined on randomly selected plants from several microboxes using a previously described protocol [6]. Briefly, leaves were washed in 100 mM phosphate buffer (pH 7) containing 0.2% Silwett by shaking for 15 minutes on a Retsch TissueLyser and sonicating for 5 minutes in a water bath. This protocol has been demonstrated to release both the epiphytic and endophytic *Pseudomonas syringae* associated with leaves [75]. Ten-fold dilution series were plated on different media. Naturally rifampicin-resistant *Variovorax* cell numbers were determined on King's B plates containing rifampicin (50 µg/ml). *Sphingomonas* cell numbers were determined on NB plates containing streptomycin (20 µg/ml). *Methylobacterium extorquens* PA1 could be distinguished from other members of the community because of its pink colony color on minimal media supplemented with 0.5% methanol. Total bacterial cell counts were determined by counting cell numbers on minimal media supplemented with 0.5% succinate.

### Statistical analysis

The R statistical environment was used for all the statistical analyses and plotting (R Development Core Team; http://www.R-project.org). Relative fluorescent intensity (RFI) was calculated by dividing individual peak area by the total peak area for each sample using the R binning script written by Ramette [38]. Parameters for the script were: a range from 500 bp to 1000 bp, a minimum RFI cutoff of 0.2%, a window size of 5 bp and shift of 1 bp. One peak was chosen to represent each species of the community (Table 1). Furthermore, RFI was normalized by the 16S rRNA gene copy number to take into account variations in copy numbers among strains. Abundance tables were analyzed using the package vegan [76]. The function vegdist with default parameters (binary = FALSE) was used to calculate the Bray-Curtis index. This function calculates the Bray-Curtis index based on proportions of different types in a sample, in contrast to the binary version of the vegdist function, which only takes into account the presence and absence of different types. Hclust was used for hierarchical clustering with the average method. The Wilcox rank sum test was used to contrast Bray-Curtis indices of comparisons between plants samples and the inoculum and within plant samples (Figure S7). Multivariate analysis of variance was conducted with the vegan functions *adonis* [77] to assess the effect of genotype and experiment on community composition (Table 2 and Table S3). To test for the effect of genotype on the RFI of each bacterial population, a generalized linear model was used with a quasibinomial distribution to correct for overdispersion. The Dunnet's test was used to test significance in the comparison to the appropriate wild-type. Multivariate analysis of variance (ANOVA) was used to test the effect of genotype and experiment on 16S rRNA gene copy numbers (Table 2 and Table S4). After normalizing to the wild-type, the numbers were log-transformed. A qqplot indicated that the standardized residuals were normally distributed; furthermore, this was confirmed by the Shapiro-Wilk test. The Student's t-Test was used to test whether each mutant differed significantly from the wild-type. Similarly, ANOVA was used to test the effect of genotype on CFU/g FW, for which the data were log10 transformed (Figure S9). The P values were adjusted for multiple testing using the Bonferroni correction.

### Nucleotide sequence accession numbers

The full-length 16S rRNA gene sequences of the *Variovorax* sp., *Arthrobacter* sp. and *Rhodococcus* sp. strains used in this study have been deposited in the European Nucleotide Archive under accession numbers HG737356, HG737357, HG737358, respectively.

## Supporting Information

Figure S1 Validation of ARISA as a semi-quantitative method. Two fold serial dilution (from 1 ng to 31.25 pg) of DNA from a *Sphingobacterium* isolate (not present in the synthetic community), was added to a DNA sample extracted from plants inoculated with the synthetic community. Community profile was determined with ARISA as described in Materials and Methods. Bacterial species abbreviations: Arthrobacter = *Arthrobacter* sp. #968, MetPA1 = *Methylobacterium extorquens* PA1, Mradio = *Methylobacterium radiotolerans* 0-1T, Rhodoccus = *Rhodococcus* sp., SphFR1 = *Sphingomonas* sp. Fr1, Sphyllo = *Sphingomonas phyllosphaerae*, Vario = *Variovorax* sp.
(PDF)

Figure S2 Time course experiment showing that the community composition changes rapidly after inoculation with the synthetic community and bacterial population increases over time. One-week-old *A. thaliana* Col0 plants were inoculated with the synthetic community, and samples were harvested immediately after spraying (t0) and 1, 2, 3, 4 weeks thereafter (respectively t1, t2, t3, t4). (a) For t0, 10 plants were pooled for each DNA sample (n = 4) and for t1, t2, t3, t4, five plant were pooled for each DNA sample (n = 4). Community profile was determined with ARISA. The Bray-Curtis dissimilarity index was used to compare communities associated with the plants (n = 4) to the inoculum (“between”) and plant samples with each other (“within”). (b and c) Population sizes were estimated on minimal media supplemented with succinate. For t0, 12 plants were pooled for each sample (n = 10); for t1, 4 plants were pooled for each sample (n = 9); and for t2, t3, t4, one plant was used for each sample (n = 12).
(PDF)

Figure S3 Reproducibility of the synthetic community. Average relative fluorescence intensity (± s.e.m) of the community-colonizing *A. thaliana* Col0 plants 2 weeks after inoculation (10 biological replicates, called “mutantA” to “mutantJ”, n = 3 or 4 technical replicates for each experiment). One-week-old *A. thaliana* Col0 plants were inoculated with the synthetic community, and samples were harvested 2 weeks after the inoculation for ARISA analyses. Bacterial species abbreviations as in Fig. S1.
(PDF)

Figure S4 Linearity of the qPCR method. (a) Two fold serial dilution (from 5 ng to 40 pg) of DNA from *Variovorax* was used as a template for qPCR with primers amplifying the 16S rRNA gene (blue diamonds). Ct, number of cycles to reach the threshold. (b) 5 ng DNA from plants grown axenically was added to the serial dilution and used as a template for qPCR with primers amplifying the 16S rRNA gene (red square) and primers amplifying the plant gene AT4G33380 (green triangles). For comparisons, the dilution series in the absence of plant DNA is shown (blue diamond). (c) The number of 16S rRNA gene copies was normalized using the plant gene.
(PDF)

Figure S5 Examples of ARISA experiments (a) Plant samples cluster in a different group than the inoculation solutions. (b–d) The mutant *lacs2* and several accession samples cluster in different group compared to wild-type and other mutant samples. Left panel: relative fluorescence intensity. Red = *Rhodococcus*, green = *Arthrobacter* sp., blue = *Variovorax* sp., yellow = *Sphingomonas* sp. Fr1, orange = *Sphingomonas phyllosphaerae*, pink = *Methylobacterium extorquens* PA1, purple = *Methylobacterium radiotolerans* 0-1T, sp. Right panel: samples are grouped by hierarchical clustering of the Bray-Curtis index (average method).
(PDF)

Figure S6 The *lacs2* and *pec1* mutants cluster in a different group than all other mutants and wild-type samples. Samples are grouped by a hierarchical clustering of the Bray-Curtis index. Each experiment is represented by a different color. The cuticle and ethylene mutants are represented by different symbols.
(PDF)

Figure S7 Different *A. thaliana* plant genotypes (55 in total) were tested for changes in bacterial community composition in independent experiments. Results of the ARISA analysis. The mean Bray-Curtis index was calculated for each genotype compared to the wild-type in pairwise comparisons (between, black bars) and for comparison of samples within each genotype (within, red dot). Genotypes are ordered by the ratio of between/within (same order than Fig. 2).
(PDF)

Figure S8 Sugar transporters do not play a role in community composition or the bacterial abundance of the synthetic community. (a) Community composition determined by an ARISA. Average relative fluorescence intensity (± s.e.m). Bacterial species abbreviations as in Fig. S1. (b) 16S rRNA gene copy number. Asterisks indicate a significant effect of genotype compared to Col-0 (*, *P*<0.05; **, *P*<0.01; ***, *P*<0.001; Bonferroni-adjusted *P* values). These experiments were repeated in triplicate with similar results.
(PDF)

Figure S9 Population sizes of different members of the community. (A) *Variovorax* cell numbers were counted on KB+rifampicin. (B) *Sphingomonas* cell numbers were counted on NB+streptomycin. (C) *Methylobacterium extorquens* PA1 cell numbers were evaluated on minimal media supplemented with methanol. (D) Total cell numbers were estimated on minimal media supplemented with succinate. Asterisks indicate a significant effect of genotype compared to Col0 (*, *P*<0.05; **, *P*<0.01; ***, *P*<0.001; Bonferroni-adjusted *P* values).
(PDF)

Figure S10 Weak effect of the *ein2* mutation for the 16S rRNA gene copy numbers. The number of 16S rRNA gene copies was normalized using a plant gene AT4G33380 and normalized to the wild-type. The number of DNA pools analyzed for each genotype is indicated in the barplot. These experiments were repeated at least in triplicate with similar results.
(PDF)

Table S1 Gammaproteobacteria strains tested in this study.
(PDF)

Table S2 *A. thaliana* mutants screened in this study.
(PDF)

Table S3 Multivariate analysis of variance for community composition.
(PDF)

Table S4 Multivariate analysis of variance for bacterial abundance.
(PDF)

Table S5 Primers used in this study.
(PDF)
