# Supplementary figures and images for: A Synthetic Community Approach Reveals Plant Genotypes Affecting the Phyllosphere Microbiota
Source: PLoS Genet. 2014 Apr 17;10(4):e1004283. doi: 10.1371/journal.pgen.1004283 (PMC3990490; doi:10.1371/journal.pgen.1004283)

Supplemental Figure 1

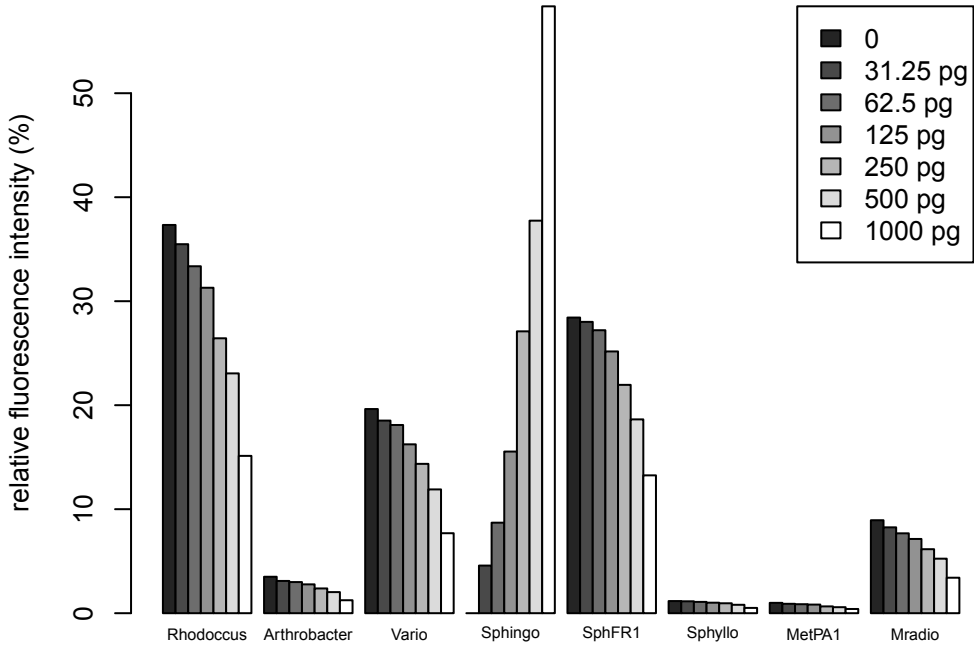

Supplement: Figure S1 — Validation of ARISA as a semi-quantitative method. Two fold serial dilution (from 1 ng to 31.25 pg) of DNA from a Sphingobacterium isolate (not present in the synthetic community), was added to a DNA sample extracted from plants inoculated with the synthetic community. Community profile was determined with ARISA as described in Materials and Methods. Bacterial species abbreviations: Arthrobacter = Arthrobacter sp. #968, MetPA1 = Methylobacterium extorquens PA1, Mradio = Methylobacterium radiotolerans 0-1T, Rhodoccus = Rhodococcus sp., SphFR1 = Sphingomonas sp. Fr1, Sphyllo = Sphingomonas phyllosphaerae, Vario = Variovorax sp. (PDF) [file pgen.1004283.s001.pdf]

Supplemental Figure 2

A

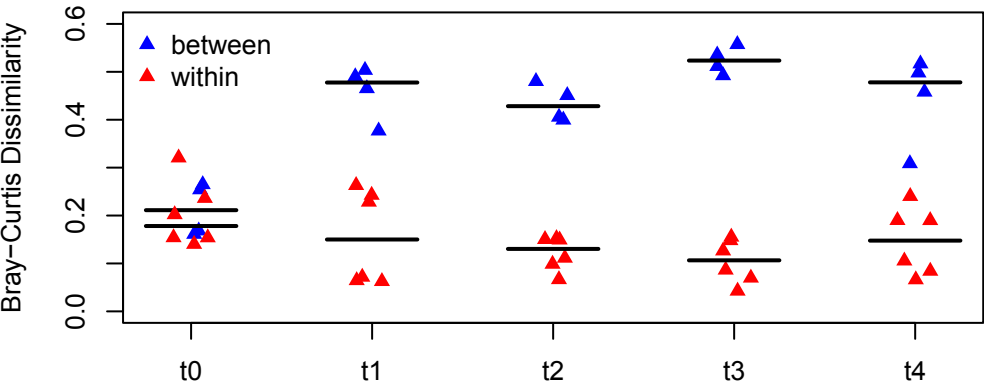

B

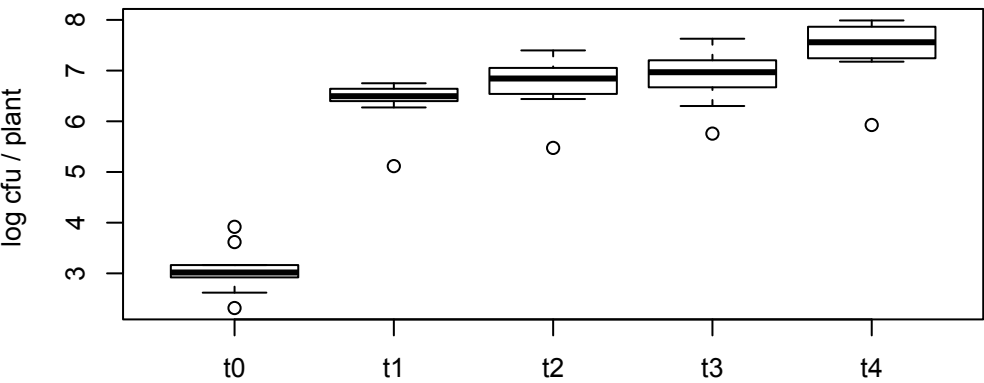

C

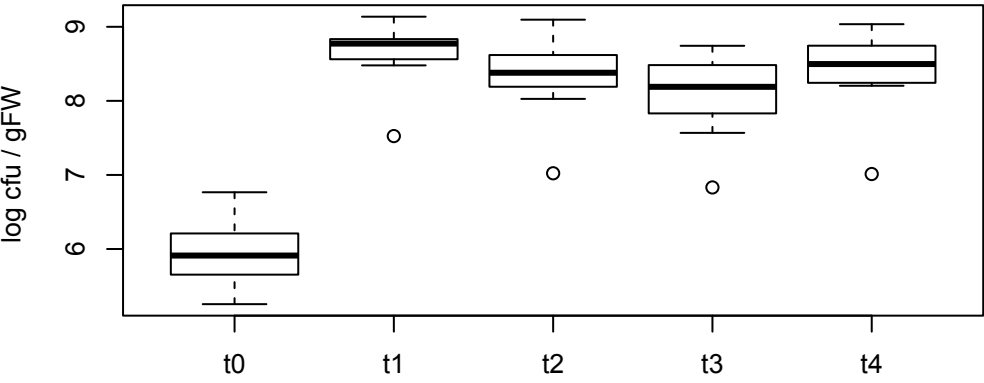

Supplement: Figure S2 — Time course experiment showing that the community composition changes rapidly after inoculation with the synthetic community and bacterial population increases over time. One-week-old A. thaliana Col0 plants were inoculated with the synthetic community, and samples were harvested immediately after spraying (t0) and 1, 2, 3, 4 weeks thereafter (respectively t1, t2, t3, t4). (a) For t0, 10 plants were pooled for each DNA sample (n = 4) and for t1, t2, t3, t4, five plant were pooled for each DNA sample (n = 4). Community profile was determined with ARISA. The Bray-Curtis dissimilarity index was used to compare communities associated with the plants (n = 4) to the inoculum (“between”) and plant samples with each other (“within”). (b and c) Population sizes were estimated on minimal media supplemented with succinate. For t0, 12 plants were pooled for each sample (n = 10); for t1, 4 plants were pooled for each sample (n = 9); and for t2, t3, t4, one plant was used for each sample (n = 12). (PDF) [file pgen.1004283.s002.pdf]

Supplemental Figure 3

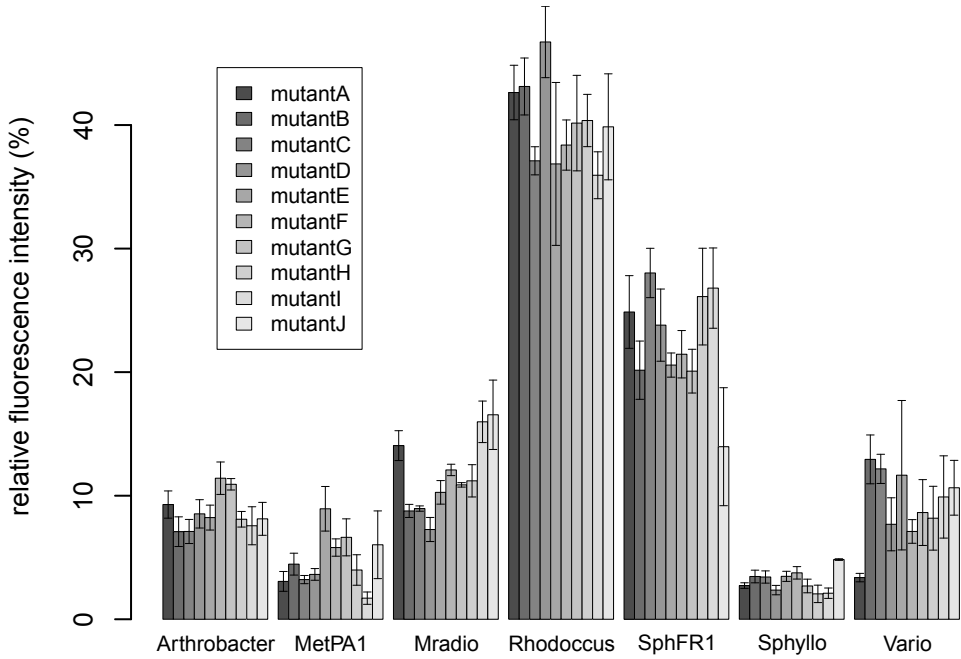

Supplement: Figure S3 — Reproducibility of the synthetic community. Average relative fluorescence intensity (± s.e.m) of the community-colonizing A. thaliana Col0 plants 2 weeks after inoculation (10 biological replicates, called “mutantA” to “mutantJ”, n = 3 or 4 technical replicates for each experiment). One-week-old A. thaliana Col0 plants were inoculated with the synthetic community, and samples were harvested 2 weeks after the inoculation for ARISA analyses. Bacterial species abbreviations as in Fig. S1. (PDF) [file pgen.1004283.s003.pdf]

## Supplemental Figure 4

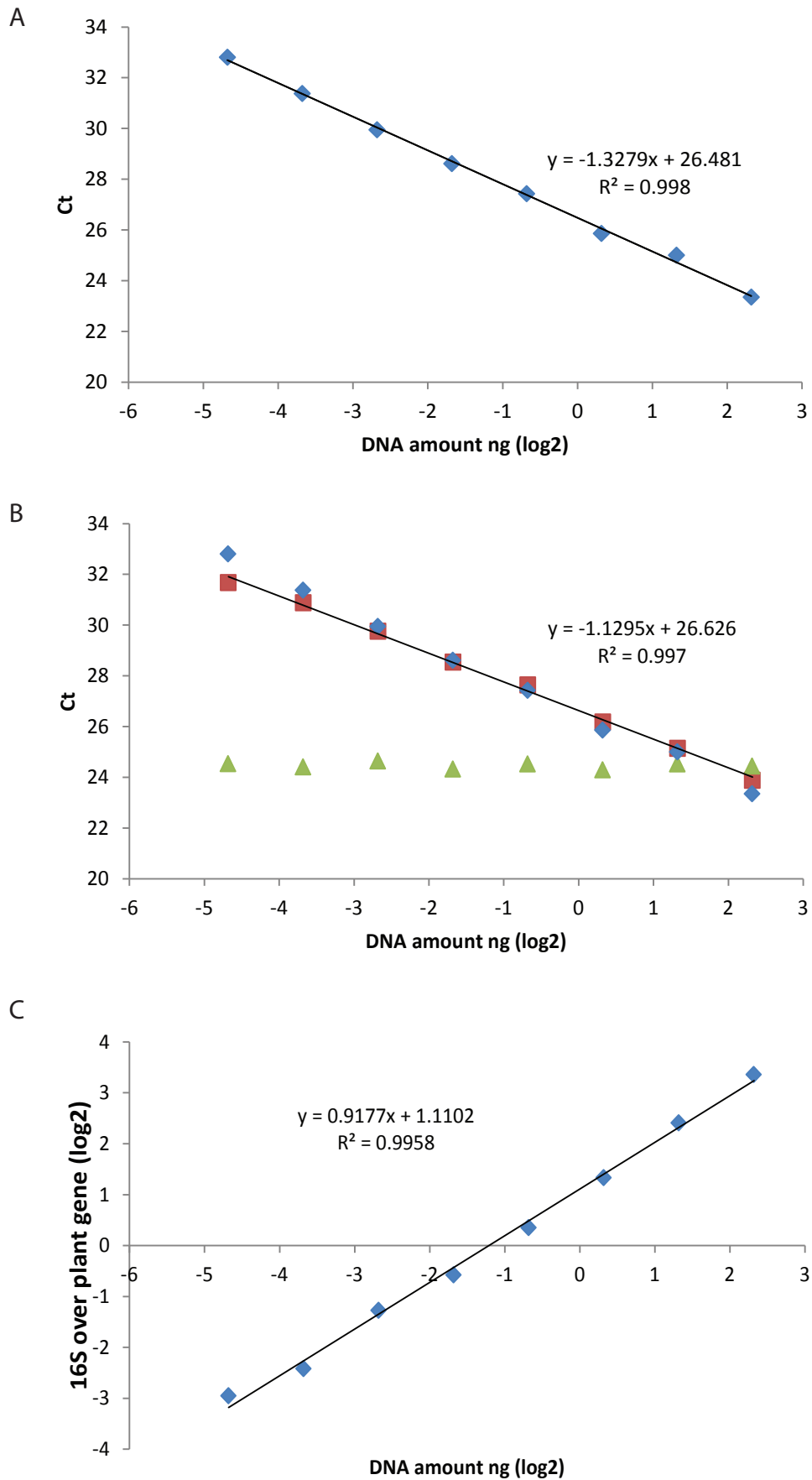

Supplement: Figure S4 — Linearity of the qPCR method. (a) Two fold serial dilution (from 5 ng to 40 pg) of DNA from Variovorax was used as a template for qPCR with primers amplifying the 16S rRNA gene (blue diamonds). Ct, number of cycles to reach the threshold. (b) 5 ng DNA from plants grown axenically was added to the serial dilution and used as a template for qPCR with primers amplifying the 16S rRNA gene (red square) and primers amplifying the plant gene AT4G33380 (green triangles). For comparisons, the dilution series in the absence of plant DNA is shown (blue diamond). (c) The number of 16S rRNA gene copies was normalized using the plant gene. (PDF) [file pgen.1004283.s004.pdf]

Supplemental Figure 5

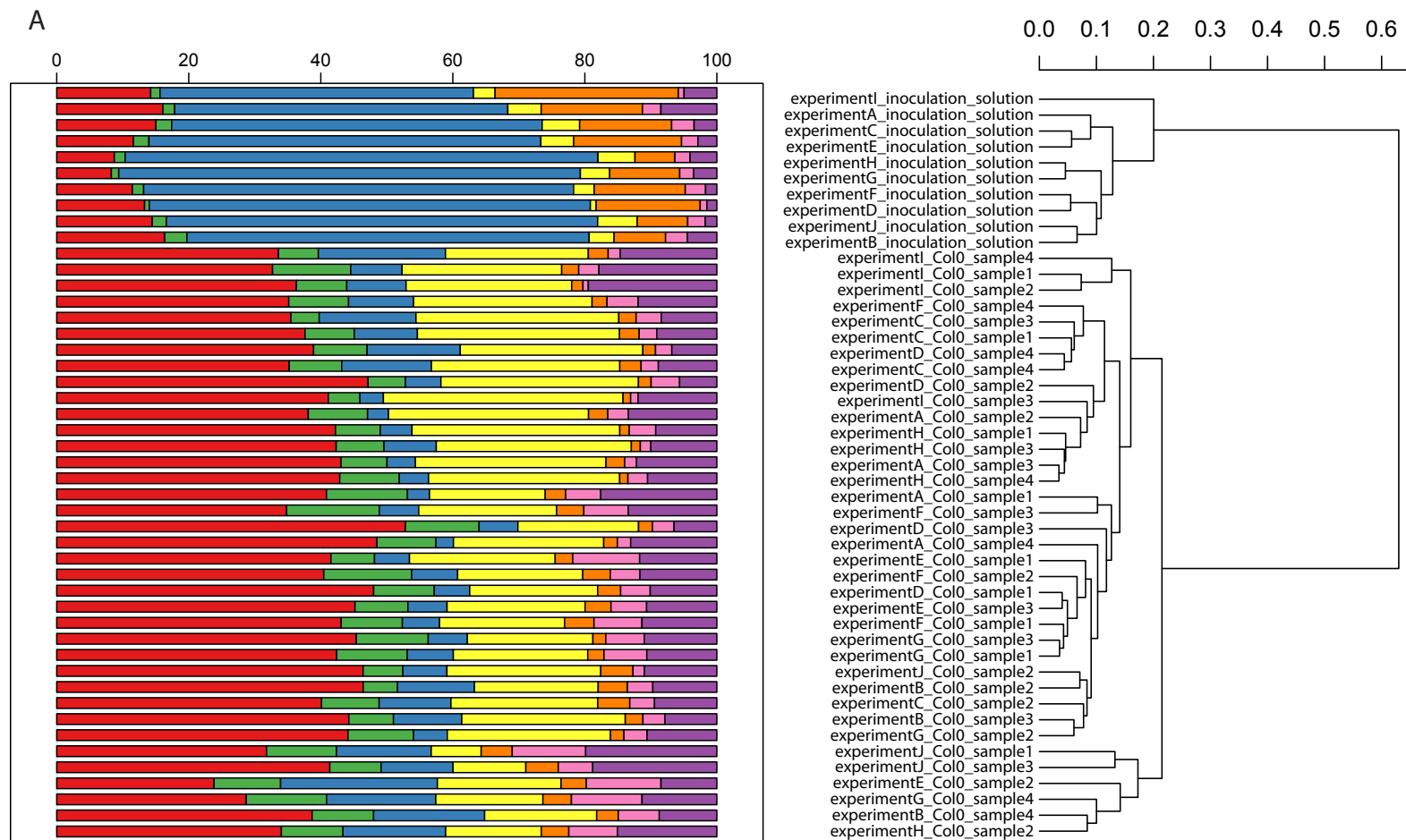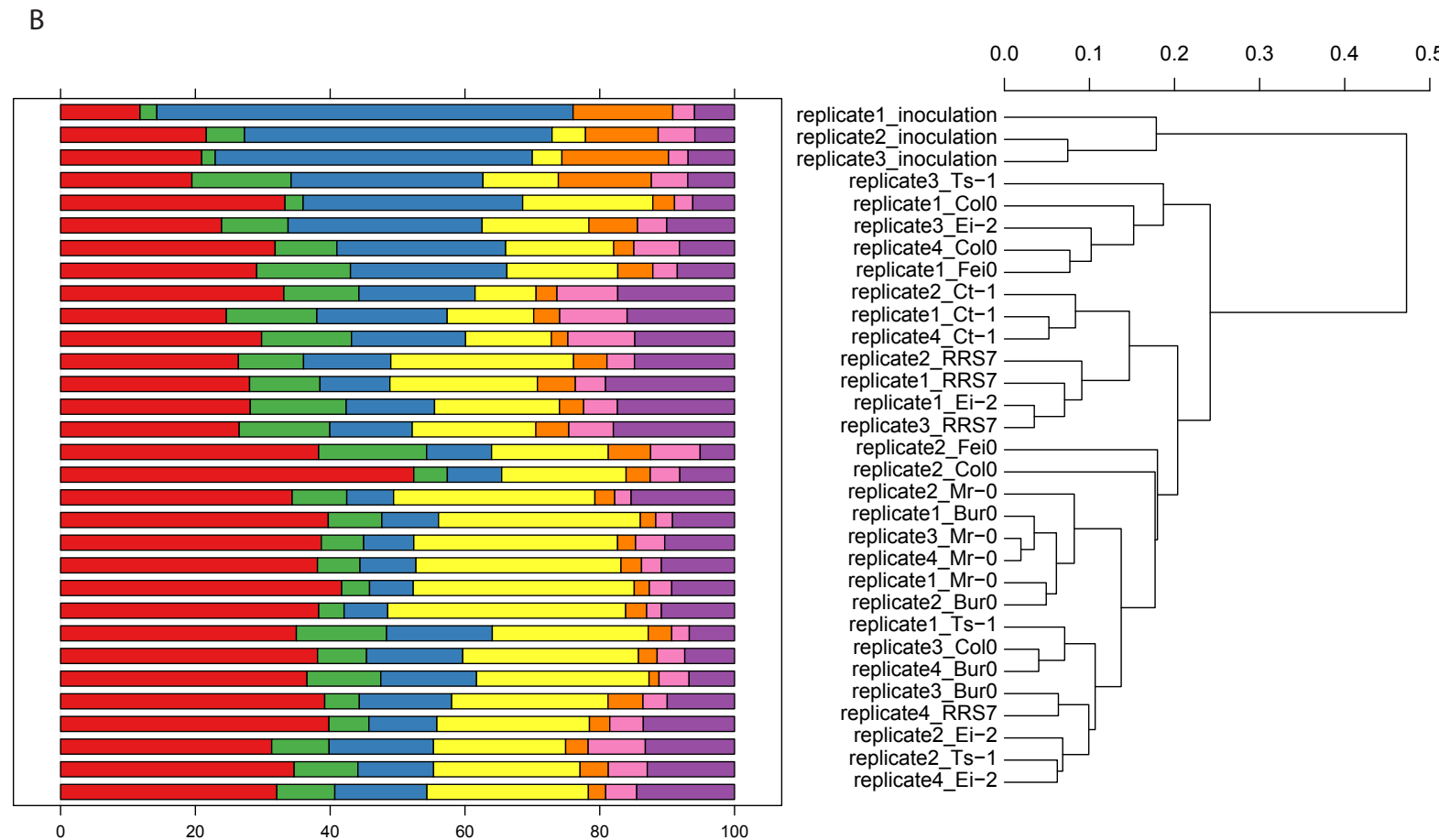

Supplemental Figure 5

C

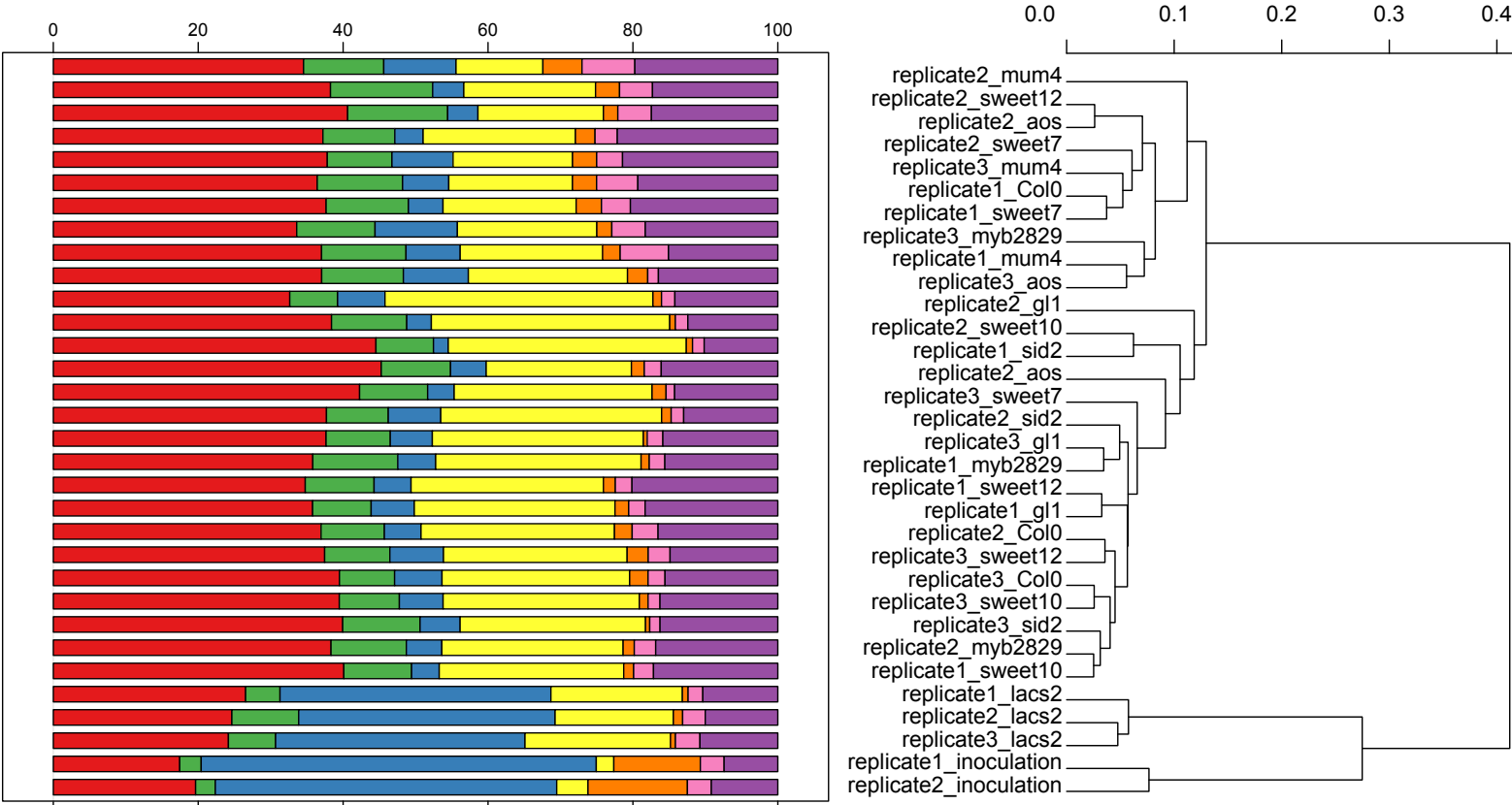

D

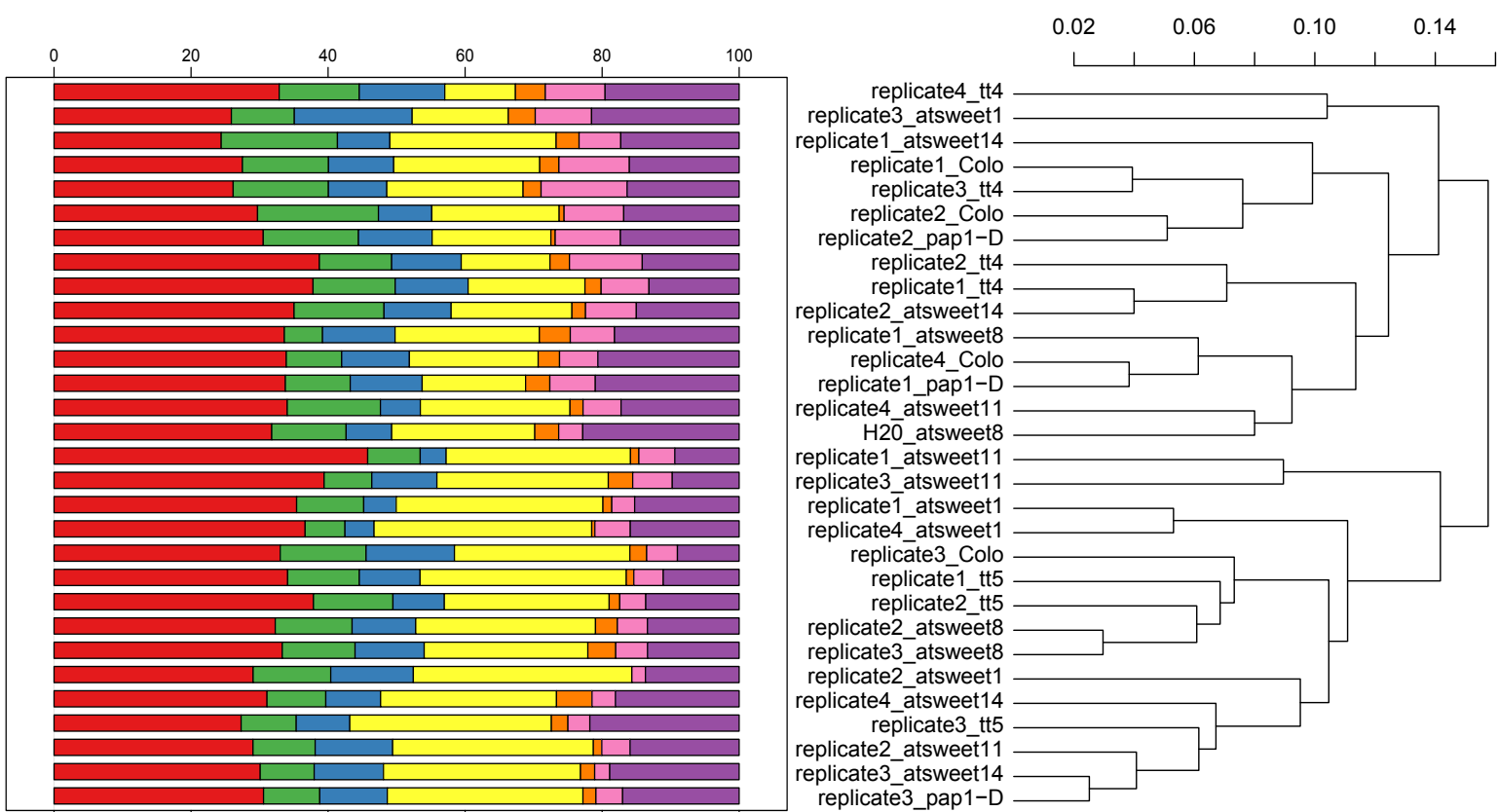

Supplement: Figure S5 — Examples of ARISA experiments (a) Plant samples cluster in a different group than the inoculation solutions. (b–d) The mutant lacs2 and several accession samples cluster in different group compared to wild-type and other mutant samples. Left panel: relative fluorescence intensity. Red = Rhodococcus, green = Arthrobacter sp., blue = Variovorax sp., yellow = Sphingomonas sp. Fr1, orange = Sphingomonas phyllosphaerae, pink = Methylobacterium extorquens PA1, purple = Methylobacterium radiotolerans 0-1T, sp. Right panel: samples are grouped by hierarchical clustering of the Bray-Curtis index (average method). (PDF) [file pgen.1004283.s005.pdf]

Supplemental Figure 7

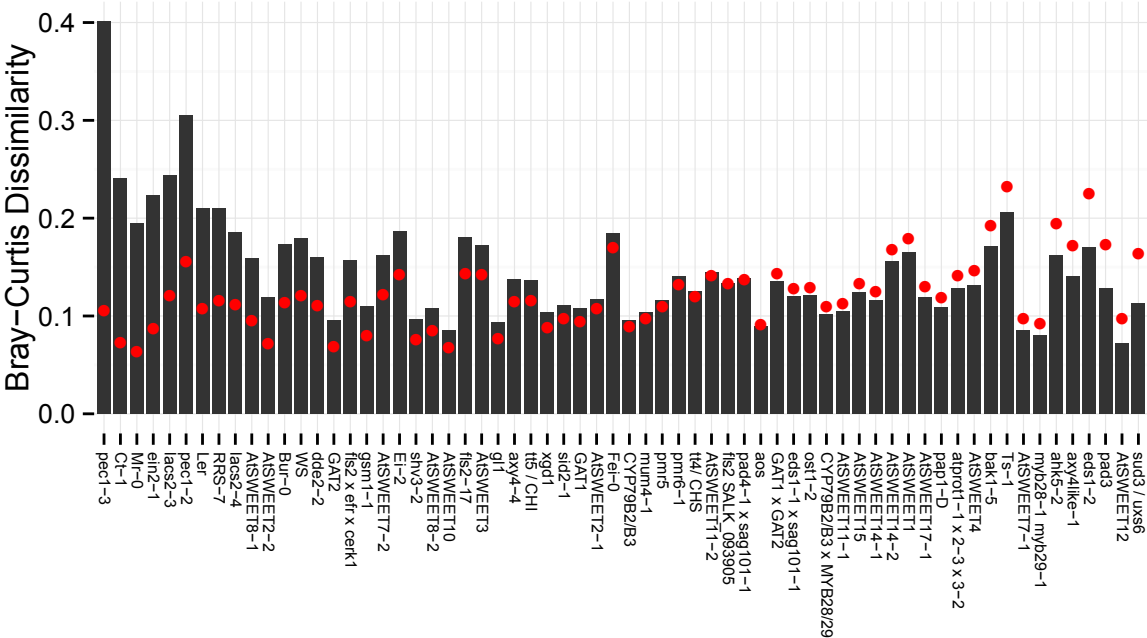

Supplement: Figure S7 — Different A. thaliana plant genotypes (55 in total) were tested for changes in bacterial community composition in independent experiments. Results of the ARISA analysis. The mean Bray-Curtis index was calculated for each genotype compared to the wild-type in pairwise comparisons (between, black bars) and for comparison of samples within each genotype (within, red dot). Genotypes are ordered by the ratio of between/within (same order than Fig. 2). (PDF) [file pgen.1004283.s007.pdf]

Supplemental Figure 8

A

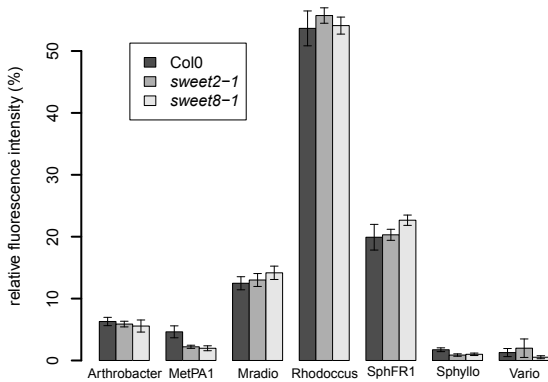

B

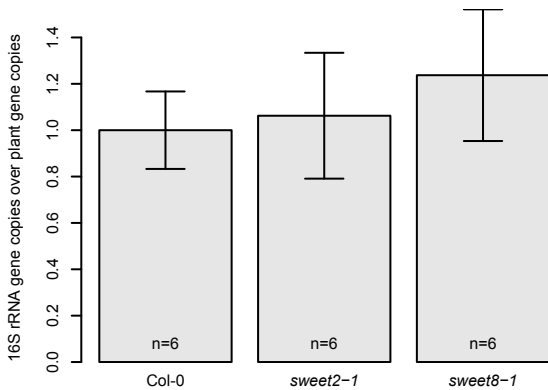

Supplement: Figure S8 — Sugar transporters do not play a role in community composition or the bacterial abundance of the synthetic community. (a) Community composition determined by an ARISA. Average relative fluorescence intensity (± s.e.m). Bacterial species abbreviations as in Fig. S1. (b) 16S rRNA gene copy number. Asterisks indicate a significant effect of genotype compared to Col-0 (*, P<0.05; **, P<0.01; ***, P<0.001; Bonferroni-adjusted P values). These experiments were repeated in triplicate with similar results. (PDF) [file pgen.1004283.s008.pdf]

Supplemental Figure 9

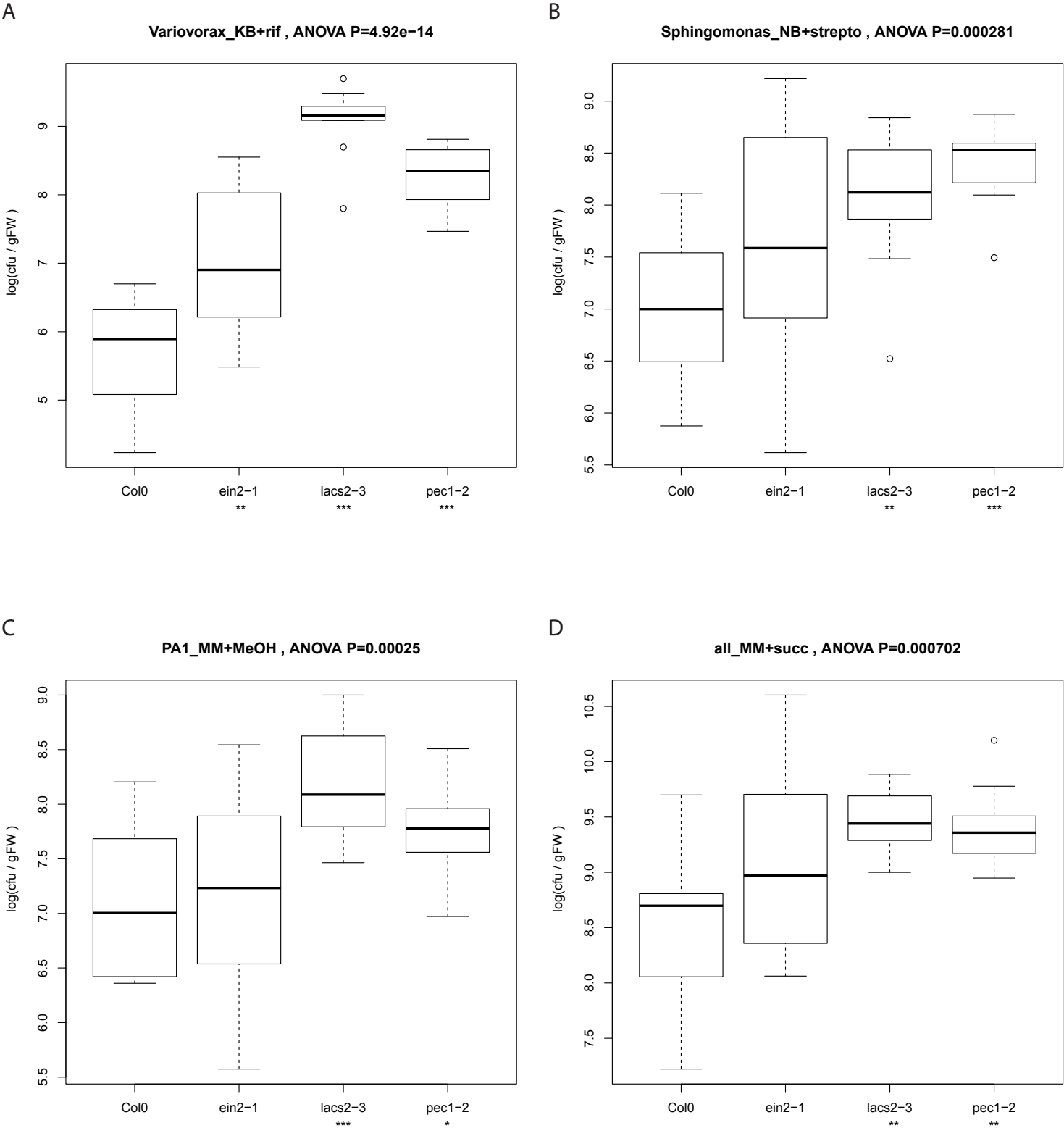

Supplement: Figure S9 — Population sizes of different members of the community. (A) Variovorax cell numbers were counted on KB+rifampicin. (B) Sphingomonas cell numbers were counted on NB+streptomycin. (C) Methylobacterium extorquens PA1 cell numbers were evaluated on minimal media supplemented with methanol. (D) Total cell numbers were estimated on minimal media supplemented with succinate. Asterisks indicate a significant effect of genotype compared to Col0 (*, P<0.05; **, P<0.01; ***, P<0.001; Bonferroni-adjusted P values). (PDF) [file pgen.1004283.s009.pdf]

## Supplemental Figure 10

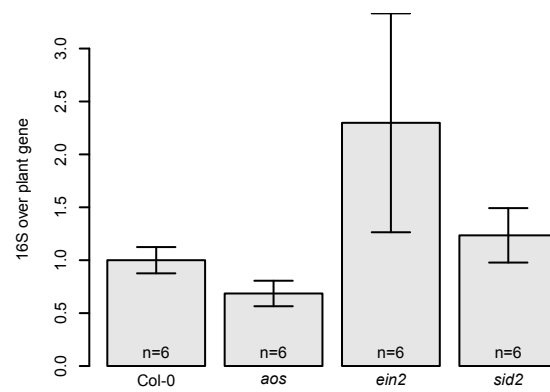

Supplement: Figure S10 — Weak effect of the ein2 mutation for the 16S rRNA gene copy numbers. The number of 16S rRNA gene copies was normalized using a plant gene AT4G33380 and normalized to the wild-type. The number of DNA pools analyzed for each genotype is indicated in the barplot. These experiments were repeated at least in triplicate with similar results. (PDF) [file pgen.1004283.s010.pdf]
