# Supplementary material for: A Synthetic Community Approach Reveals Plant Genotypes Affecting the Phyllosphere Microbiota
Source: PLoS Genet. 2014 Apr 17;10(4):e1004283. doi: 10.1371/journal.pgen.1004283 (PMC3990490; doi:10.1371/journal.pgen.1004283)
Supplement: Table S1 — Gammaproteobacteria strains tested in this study. (PDF) [file pgen.1004283.s011.pdf]

Table S1. Gammaproteobacteria strains tested in this study.

Strain	Phylum	Reference	Reason for not including in the final community used for screening
Pseudomonas syringae Psy642	Gammaproteobacteria	[78]	Reduced plant growth
Pseudomonas viridiflava	Gammaproteobacteria	Laboratory collection*	Causes disease
Pseudomonas fluorescens pf-5	Gammaproteobacteria	[79]	Reduced plant growth
Pseudomonas syringae #13	Gammaproteobacteria	Laboratory collection*	Reduced plant growth
Pantoea agglomerans 299R	Gammaproteobacteria	[80]	Causes disease

* isolated by C. Knief

References

78. Clarke CR, Cai RM, Studholme DJ, Guttman DS, Vinatzer BA (2010) *Pseudomonas syringae* strains naturally lacking the classical *P. syringae hrp/hrc* locus are common leaf colonizers equipped with an typical type III secretion system. Mol Plant Microbe Interact 23: 198-210.
79. Howell CR, Stipanovic RD (1979) Control of *Rhizoctonia solani* on cotton seedlings with *Pseudomonas fluorescens* and with an antibiotic produced by the bacterium. Phytopathology 69: 480-482.
80. Brandl M, Clark EM, Lindow SE (1996) Characterization of the indole-3 acetic acid (IAA) biosynthetic pathway in an epiphytic strain of *Erwinia herbicola* and IAA production in vitro. Can J Microb 42: 586-592.
