# Supplementary material for: A Synthetic Community Approach Reveals Plant Genotypes Affecting the Phyllosphere Microbiota
Source: PLoS Genet. 2014 Apr 17;10(4):e1004283. doi: 10.1371/journal.pgen.1004283 (PMC3990490; doi:10.1371/journal.pgen.1004283)
Supplement: Table S2 — A. thaliana mutants screened in this study. (PDF) [file pgen.1004283.s012.pdf]

Table S2. *A. thaliana* mutants screened in this study.

Mutant	AGI	Phenotype	Ref.	Seed Origin
**Cell-wall / pectin synthesis**				
axy4-4	AT1G70230	Reduced xyloglucan O-acetylation	[81]	NASC
axy4like-1	AT3G28150	Reduced xyloglucan O-acetylation	[81]	NASC
mum4-1 /rhm	At1g53500	Decrease in seed coat mucilage	[82]	NASC
pmr5	AT5G58600	Resistant to powdery mildew, enriched in pectin	[83]	NASC
pmr6-1	AT3G54920	Resistant to powdery mildew, enriched in pectin	[84]	NASC
shv3-2	AT4G26690	Ruptured root hair cells	[85]	NASC
sud3 / uxs6	At2g28760	NA	[86]	NASC
xgd1	AT5G33290	Decreased cell wall xylose	[87]	NASC
**Cuticle, trichome, stomata**				
lacs2-3, lacs2-4	AT1G49430	Permeable cuticle	[40]	Christiane Nawrath, University of Lausanne
pec1-1, pec1-4	AT2G26910	Permeable cuticle	[41]	Christiane Nawrath, University of Lausanne
ost1-2	AT4G33950	Impaired ability to close stomata in drought	[88]	Jerome Giraudat, Institut des Sciences du Végétal
gl1	AT3G27920	Glabrous (trichome-less)	[89]	Edward Farmer, University of Lausanne
**Defense signal pathways**				
ahk5-2 SALK_051626	AT5G10720	Increased susceptibility to *Pseudomonas syringae* and *Botrytis cinerea*	[90]	NASC
aos GL1	AT5G42650	Jasmonic acid (JA) deficient	[91]	Edward Farmer, University of Lausanne
dde2-2	AT5G42650	Jasmonic acid (JA) deficient	[92]	Beat Keller, University of Zurich

eds1-2	AT3G48090	Highly susceptible to Hyaloperonospora *parasitica* (downy mildew pathogen)	[93]	Jane Parker, MPI Plant Breeding
eds1xSAG101	AT3G48090<br>AT5G14930	NA	NA	Jane Parker, MPI Plant Breeding
ein2-1	AT5G03280	ethylene insensitive	[43]<br>[42]	Philippe Reymond, University of Lausanne
pad4xSAG101	AT3G52430<br>AT5G14930	Increased susceptibility to virulent *Peronospora parasitica* (oomycete)	[94]	Jane Parker, MPI Plant Breeding
sid2-1	AT1G74710	No accumulation of salicylic acid	[95]	Christiane Nawrath, University of Lausanne
**Pattern-triggered immunity**				
bak1-5	AT4G33430	Susceptible to *Pseudomonas syringae*	[96]	Cyril Zipfel, John Innes Centre, Norwich
fls2-17	AT5G46330	Flagellin insensitive	[27]	Thomas Boller, University of Basel
fls2 SALK_093905	AT5G46330	Flagellin insensitive		Akio Tani, Institute of Plant Science and Resources, Okayama
fls2xefrxcerk1	AT4G33430<br>AT2G13790<br>AT3G21630	Decreased reactive oxygen species after treatment with bacterial extract	[97]	Cyril Zipfel, the Sainsbury Laboratory, Norwich
**Secondary metabolism**				
CYP79B2/B3	AT4G39950<br>AT2G22330	Reduced abundance of indole glucosinolates	[98]	Philippe Reymond, University of Lausanne
CYP79B2/B3 x MYB28/29	At5g61420<br>At5g07690<br>AT4G39950<br>AT2G22330	Reduced abundance of aliphatic and indole glucosinolates	[99]	Philippe Reymond, University of Lausanne
gsm1-1	AT5G23010	Reduced abundance of aliphatic glucosinolates	[100,101]	Philippe Reymond, University of Lausanne

MYB28/29	At5g61420<br>At5g0769	Reduced abundance of aliphatic glucosinolates	[102]<br>[103]	Philippe Reymond, University of Lausanne
pad3	AT3G26830	Reduced abundance of camalexin	[104,105]	Philippe Reymond, University of Lausanne
pap1-D	AT1G56650	Accumulation of flavonoids including anthocyanin pigments	[106]	Philippe Reymond, University of Lausanne
tt4/ CHS (check SALK ID)	AT5G13930	Absence of anthocyanins in the leaves	[107]	Philippe Reymond, University of Lausanne
tt5 / CHI (check SALK ID)	AT3G55120	Absence of anthocyanins in the leaves	[107]	Philippe Reymond, University of Lausanne
**Transporters**				
atprot1-1 x 2-3 x 3-2	AT2G39890<br>AT3G55740<br>AT2G36590	lower uptake of glycine betaine and proline	[108]	Doris Rentsch, University of Bern
GAT1 (check SALK ID)	At1g08230	NA	[109]	Doris Rentsch, University of Bern
GAT1 x GAT2	At1g08230<br>At5g41800	NA	[109]	Doris Rentsch, University of Bern
GAT2(check SALK ID)	At5g41800	NA	[109]	Doris Rentsch, University of Bern
AtSWEET1 SALK_029479C	AT1G21460	NA	[29]	NASC
AtSWEET2-1 SALK_048430C<br>*AtSWEET2-2 SALK_034060C*	AT3G14770	NA	[29]	NASC
AtSWEET3 SALK_124142C	AT5G53190	NA	[29]	NASC
AtSWEET4 SALK_072225	AT3G28007	NA	[29]	NASC
AtSWEET7-1 SALK_071508C<br>*AtSWEET7-2 SAIL_1233_D07*	AT4G10850	NA	[29]	NASC
AtSWEET8-1 SALK_142803C<br>*AtSWEET8-2 SALK_092239C*	AT5G40260	Reduced male fertility	[29,110]	NASC
AtSWEET10 SALK_144826	AT5G50790	NA	[29]	NASC

AtSWEET11-1 SALK_073269C<br>*AtSWEET11-2 SALK_095891C*	AT3G48740	NA	[29]	NASC
AtSWEET12 SALK_077789C	AT5G23660	NA	[29]	NASC
AtSWEET14-1 GK-113H08<br>*AtSWEET14-2 SALK_010224C*	AT4G25010	NA	[29]	NASC
AtSWEET15 SALK_031720C	AT5G13170	Less sensitive to high salinity	[29,111]	NASC
AtSWEET17 SALK_012485C	AT4G15920	NA	[29]	NASC
**Accessions**				
Bur0, Ct-1, Ei-2, Fei0, Ler, Mr-0, RRS-7, Ts-1, WS				Philippe Reymond, University of Lausanne

References

81. Gille S, de Souza A, Xiong GY, Benz M, Cheng K, et al. (2011) O-Acetylation of *Arabidopsis* hemicellulose xyloglucan requires AXY4 or AXY4L, proteins with a TBL and DUF231 domain. Plant Cell 23: 4041-4053.
82. Western TL, Young DS, Dean GH, Tan WL, Samuels AL, et al. (2004) MUCILAGE-MODIFIED4 encodes a putative pectin biosynthetic enzyme developmentally regulated by APETALA2, TRANSPARENT TESTA GLABRA1, and GLABRA2 in the *Arabidopsis* seed coat. Plant Physiol 134: 296-306.
83. Vogel JP, Raab TK, Somerville CR, Somerville SC (2004) Mutations in PMR5 result in powdery mildew resistance and altered cell wall composition. Plant J 40: 968-978.
84. Vogel JP, Raab TK, Schiff C, Somerville SC (2002) PMR6, a pectate lyase-like gene required for powdery mildew susceptibility in *Arabidopsis*. Plant Cell 14: 2095-2106.
85. Hayashi S, Ishii T, Matsunaga T, Tominaga R, Kuromori T, et al. (2008) The glycerophosphoryl diester phosphodiesterase-like proteins SHV3 and its homologs play important roles in cell wall organization. Plant Cell Physiol 49: 1522-1535.
86. Usadel B (2004) Untersuchungen zur Biosynthese der Zellwand: Universität Potsdam.
87. Jensen JK, Sorensen SO, Harholt J, Geshi N, Sakuragi Y, et al. (2008) Identification of a xylogalacturonan xylosyltransferase involved in pectin biosynthesis in *Arabidopsis*. Plant Cell 20: 1289-1302.
88. Mustilli AC, Merlot S, Vavasseur A, Fenzi F, Giraudat J (2002) *Arabidopsis* OST1 protein kinase mediates the regulation of stomatal aperture by abscisic acid and acts upstream of reactive oxygen species production. Plant Cell 14: 3089-3099.
89. Herman PL, Marks MD (1989) Trichome development in *Arabidopsis thaliana*. II. Isolation and complementation of the GLABROUS1 gene. Plant Cell 1: 1051-1055.
90. Pham J, Liu J, Bennett MH, Mansfield JW, Desikan R (2012) *Arabidopsis* histidine kinase 5 regulates salt sensitivity and resistance against bacterial and fungal infection. New Phytologist 194: 168-180.
91. Park JH, Halitschke R, Kim HB, Baldwin IT, Feldmann KA, et al. (2002) A knock-out mutation in allene oxide synthase results in male sterility and defective wound signal transduction in *Arabidopsis* due to a block in jasmonic acid biosynthesis. Plant J 31: 1-12.
92. von Malek B, van der Graaff E, Schneitz K, Keller B (2002) The *Arabidopsis* male-sterile mutant *dde2-2* is defective in the ALLENE OXIDE SYNTHASE gene encoding one of the key enzymes of the jasmonic acid biosynthesis pathway. Planta 216: 187-192.
93. Falk A, Feys BJ, Frost LN, Jones JDG, Daniels MJ, et al. (1999) EDS1, an essential component of R gene-mediated disease resistance in *Arabidopsis* has homology to eukaryotic lipases. Proc Natl Acad Sci U S A 96: 3292-3297.
94. Feys BJ, Wiermer M, Bhat RA, Moisan LJ, Medina-Escobar N, et al. (2005) *Arabidopsis* SENESCENCE-ASSOCIATED GENE101 stabilizes and signals within an ENHANCED DISEASE SUSCEPTIBILITY1 complex in plant innate immunity. Plant Cell 17: 2601-2613.
95. Nawrath C, Metraux JP (1999) Salicylic acid induction-deficient mutants of *Arabidopsis* express PR-2 and PR-5 and accumulate high levels of camalexin after pathogen inoculation. Plant Cell 11: 1393-1404.
96. Roux M, Schwessinger B, Albrecht C, Chinchilla D, Jones A, et al. (2011) The *Arabidopsis* leucine-rich repeat receptor-like kinases BAK1/SERK3 and BKK1/SERK4 are required for innate immunity to hemibiotrophic and biotrophic pathogens. Plant Cell 23: 2440-2455.

97. Gimenez-Ibanez S, Ntoukakis V, Rathjen JP (2009) The LysM receptor kinase CERK1 mediates bacterial perception in *Arabidopsis*. Plant Signal Behav 4: 539-541.
98. Zhao Y, Christensen SK, Fankhauser C, Cashman JR, Cohen JD, et al. (2001) A role for flavin monooxygenase-like enzymes in auxin biosynthesis. Science 291: 306-309.
99. Schweizer F (2012) Transcriptions factors involved in plant defence against herbivores in *Arabidopsis thaliana*: Lausanne University, Switzerland.
100. Haughn GW, Davin L, Giblin M, Underhill EW (1991) Biochemical genetics of plant secondary metabolites in *Arabidopsis thaliana* - the glucosinolates. Plant Physiol 97: 217-226.
101. Kroymann J, Textor S, Tokuhisa JG, Falk KL, Bartram S, et al. (2001) A gene controlling variation in *Arabidopsis* glucosinolate composition is part of the methionine chain elongation pathway. Plant Physiol 127: 1077-1088.
102. Beekwilder J, van Leeuwen W, van Dam NM, Bertossi M, Grandi V, et al. (2008) The impact of the absence of aliphatic glucosinolates on insect herbivory in *Arabidopsis*. PLoS One 3: e2068.
103. Sonderby IE, Hansen BG, Bjarnholt N, Ticconi C, Halkier BA, et al. (2007) A systems biology approach identifies a R2R3 MYB gene subfamily with distinct and overlapping functions in regulation of aliphatic glucosinolates. PLoS One 2: e1322.
104. Glazebrook J, Ausubel FM (1994) Isolation of phytoalexin-deficient mutants of *Arabidopsis thaliana* and characterization of their interactions with bacterial pathogens. Proc Natl Acad Sci U S A 91: 8955-8959.
105. Schuhegger R, Nafisi M, Mansourova M, Petersen BL, Olsen CE, et al. (2006) Cyp71b15 (Pad3) catalyzes the final step in camalexin biosynthesis. Plant Physiology 141: 1248-1254.
106. Borevitz JO, Xia Y, Blount J, Dixon RA, Lamb C (2000) Activation tagging identifies a conserved MYB regulator of phenylpropanoid biosynthesis. Plant Cell 12: 2383-2394.
107. Shirley BW, Kubasek WL, Storz G, Bruggemann E, Koornneef M, et al. (1995) Analysis of *Arabidopsis* mutants deficient in flavonoid biosynthesis. Plant J 8: 659-671.
108. Lehmann S, Gumy C, Blatter E, Boeffel S, Fricke W, et al. (2011) In planta function of compatible solute transporters of the AtProT family. J Exp Bot 62: 787-796.
109. Meyer A, Eskandari S, Grallath S, Rentsch D (2006) AtGAT1, a high affinity transporter for gamma-aminobutyric acid in *Arabidopsis thaliana*. J Biol Chem 281: 7197-7204.
110. Guan YF, Huang XY, Zhu J, Gao JF, Zhang HX, et al. (2008) RUPTURED POLLEN GRAIN1, a member of the MtN3/saliva gene family, is crucial for exine pattern formation and cell integrity of microspores in *Arabidopsis*. Plant Physiol 147: 852-863.
111. Seo PJ, Park JM, Kang SK, Kim SG, Park CM (2011) An *Arabidopsis* senescence-associated protein SAG29 regulates cell viability under high salinity. Planta 233: 189-200.
112. Ito H, Iizuka H (1971) Taxonomic studies on a radio-resistant *Pseudomonas*. XII. Studies on microorganisms of cereal grain. Agricultural Biol Chem 35: 1566-1571.
113. Rivas R, Abril A, Trujillo ME, Velazquez E (2004) *Sphingomonas phyllosphaerae* sp nov., from the phyllosphere of *Acacia* caven in Argentina. Int J System Evol Microbiol 54: 2147-2150.
