# Supplementary material for: A Synthetic Community Approach Reveals Plant Genotypes Affecting the Phyllosphere Microbiota
Source: PLoS Genet. 2014 Apr 17;10(4):e1004283. doi: 10.1371/journal.pgen.1004283 (PMC3990490; doi:10.1371/journal.pgen.1004283)
Supplement: Table S3 — Multivariate analysis of variance for community composition. (PDF) [file pgen.1004283.s013.pdf]

Table S3. Multivariate analysis of variance for community composition.

ADONIS	*lacs2-3*	*pec1-3*	*ein2*	Ct-1	Ler	Mr-0	RRS-7
Experiment	0.021	0.002	0.001	0.001	0.001	0.001	0.001
Experiment* genotype	0.110	0.060	0.66	0.106	0.001	0.001	0.002
Genotype across experiment	0.001*	0.001*	0.001*	0.001*	0.001*	0.001*	0.001*
**Difference between the other genotype and Col0**							
Within experiment 1	0.088	0.024<br>Figure 3A	0.023	0.036	0.025	0.029	0.03
Within experiment 2	0.022<br>Figure 3A	0.037	0.12	0.004	0.019	0.007	0.584
Within experiment 3	0.001	0.003	0.034<br>Figure 3B	0.009<br>Figure 5A	0.002<br>Figure 5A	0.005<br>Figure 5A	0.003<br>Figure 5A
Within experiment 4			0.151				

Values shown are the *P* values resulting from analysis of variance using distance matrices (adonis) of community composition associated with leaves of Col0 and the indicated genotype in the replicate experiments (3 or 4 experiments depending on the genotype). Values for within an experiment are *P* values from adonis. Asterisks mark tests for ‘Genotype’ that are significant after Bonferroni correction for multiple testing (seven independent tests).
