# Supplementary material for: A Synthetic Community Approach Reveals Plant Genotypes Affecting the Phyllosphere Microbiota
Source: PLoS Genet. 2014 Apr 17;10(4):e1004283. doi: 10.1371/journal.pgen.1004283 (PMC3990490; doi:10.1371/journal.pgen.1004283)
Supplement: Table S4 — Multivariate analysis of variance for bacterial abundance. (PDF) [file pgen.1004283.s014.pdf]

Table S4. Multivariate analysis of variance for bacterial abundance.

ANOVA	*lacs2-3*	*pec1-3*	*ein2*	Ct-1	Ler	Mr-0	RRS-7
Experiment	0.8926	0.03544	0.65150	0.7120	0.08908	5.731e-05	0.1398
Experiment* genotype	0.8640	0.06663	0.46089	0.6932	0.13805	5.889e-05	0.1505
Genotype across experiment	2.935e-06*	5.085e-05*	0.00518*	2.711e-06*	1.033e-07*	4.802e-10*	2.099e-09*
**Difference between the other genotype and Col0**							
Within experiment 1	0.01865	0.2093	0.0435	0.006795	0.01193	0.8894	0.004711
Within experiment 2	0.00491	0.2321	0.2311	0.01114	0.01185	1.087e-05	0.009494
Within experiment 3	0.01125	8.501e-05	0.6296	0.01429	0.0004356	2.808e-05	1.288e-06
Within experiment 4			0.2664				

Values shown are the P values resulting from analysis of variance ANOVA of the 16S rRNA copy numbers of Col0 and the indicated genotype in the replicate experiments 3 or 4 experiments depending on the genotype. Data were first normalized to the wild-type and then log-transformed. Values for within an experiment are P values from a student *t- test* unequal variance. Asterisks mark tests for 'Genotype' that are significant after Bonferroni correction for multiple testing seven independent tests.
