# Supplementary material for: A Synthetic Community Approach Reveals Plant Genotypes Affecting the Phyllosphere Microbiota
Source: PLoS Genet. 2014 Apr 17;10(4):e1004283. doi: 10.1371/journal.pgen.1004283 (PMC3990490; doi:10.1371/journal.pgen.1004283)
Supplement: Table S5 — Primers used in this study. (PDF) [file pgen.1004283.s015.pdf]

Table S5. Primers used in this study.

Primer name	Primer sequence	Reference
1492F	AAGTCGTAACAAGGTARCCGTA	[69]
23Sr	GGGTTBCCCCATTCRG	[70]
799f	AACMGGATTAGATACCCKG	[71]
904R	CCCCGTCAATTCITTTGAGTTTYAR	[72]
ExpF	ATACAGAAACAACCACCCAAAAG	Madlen Vetter, University of Chicago
ExpR	ACGGTACTCCAATTTTCAAGACTC	Madlen Vetter, University of Chicago
