# Supplementary material for: A Synthetic Community Approach Reveals Plant Genotypes Affecting the Phyllosphere Microbiota
Source: PLoS Genet. 2014 Apr 17;10(4):e1004283. doi: 10.1371/journal.pgen.1004283 (PMC3990490; doi:10.1371/journal.pgen.1004283)

Supplemental Figure 6

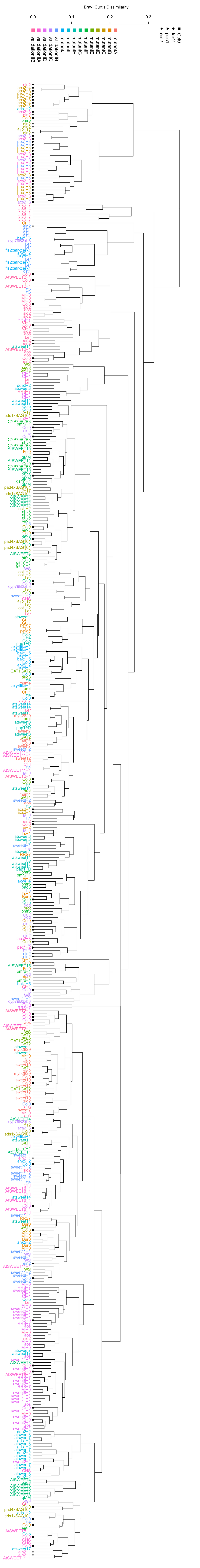

Supplement: Figure S6 — The lacs2 and pec1 mutants cluster in a different group than all other mutants and wild-type samples. Samples are grouped by a hierarchical clustering of the Bray-Curtis index. Each experiment is represented by a different color. The cuticle and ethylene mutants are represented by different symbols. (PDF) [file pgen.1004283.s006.pdf]
